# Integrating extracellular vesicle and circulating cell‐free DNA analysis using a single plasma aliquot improves the detection of HER2 positivity in breast cancer patients

**DOI:** 10.1002/jex2.108

**Published:** 2023-09-25

**Authors:** Vera Mugoni, Yari Ciani, Orsetta Quaini, Simone Tomasini, Michela Notarangelo, Federico Vannuccini, Alessia Marinelli, Elena Leonardi, Stefano Pontalti, Angela Martinelli, Daniele Rossetto, Isabella Pesce, Sheref S. Mansy, Mattia Barbareschi, Antonella Ferro, Orazio Caffo, Gerhardt Attard, Dolores Di Vizio, Vito Giuseppe D'Agostino, Caterina Nardella, Francesca Demichelis

**Affiliations:** ^1^ Department of Cellular, Computational and Integrative Biology University of Trento Trento Italy; ^2^ Unit of Surgical Pathology, Santa Chiara Hospital, APSS Trento Italy; ^3^ Department of Medical Oncology Santa Chiara Hospital, APSS Trento Italy; ^4^ University College London Cancer Institute London UK; ^5^ Department of Surgery, Division of Cancer Biology and Therapeutics Cedars‐Sinai Medical Center Los Angeles California USA

**Keywords:** breast cancer, cell‐free DNA (cfDNA), ddPCR, EV‐RNA, extracellular vesicles (EV), HER2, ImageStream, ONCE approach

## Abstract

Multi‐analyte liquid biopsies represent an emerging opportunity for non‐invasive cancer assessment. We developed ONCE (One Aliquot for Circulating Elements), an approach for the isolation of extracellular vesicles (EV) and cell‐free DNA (cfDNA) from a single aliquot of blood. We assessed ONCE performance to classify HER2‐positive early‐stage breast cancer (BrCa) patients by combining EV‐associated RNA (EV‐RNA) and cfDNA signals on *n* = 64 healthy donors (HD) and non–metastatic BrCa patients. Specifically, we isolated EV‐enriched samples by a charge‐based (CB) method and investigated EV‐RNA and cfDNA by next‐generation sequencing (NGS) and by digital droplet PCR (ddPCR). Sequencing of cfDNA and EV‐RNA from HER2‐ and HER2+ patients demonstrated concordance with in situ molecular analyses of matched tissues. Combined analysis of the two circulating analytes by ddPCR showed increased sensitivity in ERBB2/HER2 detection compared to single nucleic acid components. Multi‐analyte liquid biopsy prediction performance was comparable to tissue‐based sequencing results from TCGA. Also, imaging flow cytometry analysis revealed HER2 protein on the surface of EV isolated from the HER2+ BrCa plasma, thus corroborating the potential relevance of studying EV as companion analyte to cfDNA. This data confirms the relevance of combining cfDNA and EV‐RNA for HER2 cancer assessment and supports ONCE as a valuable tool for multi‐analytes liquid biopsies’ clinical implementation.

## 1 INTRODUCTION

Liquid biopsies have emerged as a promising non‐invasive tool to facilitate real‐time detection and molecular profiling of primary and metastatic tumors (Snow et al., 2019). Tests based on cell‐free DNA (cfDNA) have been shown to hold diagnostic and prognostic significance in a range of cancer types (Pascual et al., 2022), including BrCa (Fernandez‐Garcia et al., 2019; Phallen et al., 2017). However, the sole analysis of cfDNA misses the information associated with gene expression, thereby limiting the potential utility of liquid biopsies.

Circulating materials actively investigated as transcriptomic resources include the RNA carried by circulating tumor cells (CTCs) and RNA circulating in the blood as free molecules or associated with extracellular vesicles (EV) and non‐vesicular extracellular nanoparticles, such as lipoproteins (Li et al., 2018), ribonucleoproteins (LaPlante et al., 2023) and supermeres (Zhang et al., 2021). Specifically, the RNA cargo of EV contains a variety of high‐quality RNA species, including protein‐coding transcripts (Amorim et al., 2017; Cufaro et al., 2019) and offers the compelling advantage of being protected from degradation by the EV lipid bilayer and of being overall more abundant than CTCs‐associated RNA. Further, CTCs are extremely rare in early non‐metastatic disease states (Heidrich et al., 2021). Analysis by Next Generation Sequencing (NGS) or high‐throughput technologies, such as quantitative real‐time PCR (qRT‐PCR), have already demonstrated the utility of EV‐RNA for the detection of cancer–associated alterations in advanced BrCa (Conley et al., 2017; Xu et al., 2018). Furthermore, EV represent an attractive circulating component for liquid biopsy tests as they act as carriers for multiple proteins, including cancer biomarkers such as the HER2 receptor in the context of BrCa (Hofmann et al., 2020; Li et al., 2021; Nanou et al., 2020; Tian et al., 2021).

The integrated analysis of circulating genomic and transcriptomic information represents an attractive advancement for multi‐analytes liquid biopsies as attempted by innovative company's proprietary platforms such as the ExoLution Plus (Mohrmann et al., 2018) in the framework of ExoDx EGFR test of non‐small cell lung cancer patients (Castellanos‐Rizaldos et al., 2019).

We, therefore, sought to address the challenge of implementing the analysis of circulating genomic and transcriptomic information by establishing a novel, easily reproducible assay, implemented on a single plasma aliquot, that can provide useful information from both plasma DNA and EV and could be adapted with commonly utilized laboratory procedures. Our assay is named ONCE (ONe Aliquot for Circulating Elements), and represents the first non‐commercial method to isolate EV and cfDNA from a single plasma aliquot. We built ONCE on commonly utilized isolation methodologies that enrich for EV, such as ultracentrifugation (UC), size exclusion chromatography (SEC), and a charge ‐based (CB) method (Thery et al., 2018). However, it is recognized that EV‐enriched samples include co‐isolated nanoparticles of similar size and/or charge (i.e., low and high‐density lipoproteins) (Holcar et al., 2021).

Here, we provide a proof‐of‐principle validation of EV‐RNA and cfDNA isolated through ONCE as informative analytes for detecting the *ERBB2*/HER2 biomarker in a cohort of early‐stage breast cancer (BrCa) patients with tissue‐defined HER2 status.

Overall, our work implements a multimodal approach for multi‐analytes liquid biopsies on a single aliquot of plasma, ultimately leading to improved detection sensitivity of cancer cargo at the early stages of the disease.

## 2 MATERIALS AND METHODS

### 2.1 Experimental design and human sample collection

Blood samples from HDs were collected on a protocol approved by the University of Trento Ethics Committee (ID # 2017‐010). BrCa patients’ plasma samples were prospectively collected on a protocol approved by the Ethics Committee of Santa Chiara Hospital in Trento (Rep.Int.12315 of 24 July 2017) with written informed consent. The experimental design was realized as described in Supplementary Materials and Methods.

### 2.2 Blood processing and plasma isolation

Whole peripheral blood (8.0 –10 mL) was collected into K2EDTA‐containing tubes (Vacuette, Greiner Bio‐One) or Streck tubes (Streck Cell‐Free DNA BCT; Streck; La Vista, NE, USA) for comparison. Plasma from K2EDTA tubes was isolated within 2 h from the collection by two‐step centrifugation at room temperature (1700 rcf × 15 min; 3000 rcf × 10 min). Plasma from Streck tubes was isolated within 5 days from the collection by two‐step centrifugation at room temperature (1700 rcf × 15 min; 15,000 rcf × 20 min). After isolation, the plasma obtained from the starting volume of blood was immediately processed or aliquoted (1.8 mL of plasma/vial) and stored at −80°C. For the application of the ONCE approach, we utilized one 1.8 mL aliquot of plasma.

### 2.3 ONCE protocol: Combined isolation of EV and cfDNA from human plasma

#### 2.3.1 EV isolation by charge‐based (CB) method and cfDNA extraction

The human plasma samples were filtered (Minisart NML syringe filters, pore size: 0.8 μm; Sartorius) and diluted with 1X phosphate‐buffered saline without calcium and magnesium buffer (Gibco) at 1:3 (v/v) ratio and processed for EV isolation by a charge—based isolation method as described before (Notarangelo et al., 2020, 2019). The diluted plasma was recovered and processed for cfDNA isolation by QIAmp Circulating Nucleic Acid kit (Qiagen) (Lee et al., 2018).

#### 2.3.2 EV isolation by ultracentrifugation (UC) and cell‐free DNA (cfDNA) extraction

EV were separated by UC on an Optima MAX‐XP ultracentrifuge (Beckman Coulter) equipped with a TLA55 rotor. The plasma samples were filtered (Minisart NML syringe filters, pore size: 0.8 μm; Sartorius) and cleaned from cell debris by two serial centrifugation steps: 2000 × *g* for 10 min and 10.000 × *g* for 20 min. EV were then pelleted at 100.000 g for 70 min, washed with 1 mL of 1xPBS (Gibco), and re‐pelleted at 100.000 g for 70 min as previously reported (Thery et al., 2006). The liquid fractions (plasma and washing PBS) recovered from the UC steps were pooled in a separate clean tube and processed for cfDNA isolation as previously described (Lee et al., 2018).

#### 2.3.3 EV isolation by size exclusion chromatography (SEC) (Izon qEV2 columns) and cfDNA extraction

Plasma samples were filtered (Minisart NML syringe filters, pore size: 0.8 μm; Sartorius) and cleaned from debris by two serial centrifugation steps: 1.500 × *g* for 10 min followed by 10.000 × *g* for 10 min before loading on a pre‐rinsed qEV2/70 nm column (Izon Science LTD, Cat. No. SP4). EV were collected from 1st to 5th fractions by using an Izon Automatic Fraction Collector (AFC) and concentrated in Amicon Ultra 15 filtering units (Merck Millipore, Cat. No. UFC910024) by centrifugation at 3.000 × *g* for 20 min on a 5810R benchtop centrifuge (Eppendorf). The volumes eluted between the 6th and the 20th fractions were collected and processed for cfDNA isolation as previously described (Lee et al., 2018).

### 2.4 Quantitation of EV

The size and concentration of the EV were quantitated using Tunable Resistive Pulse Sensing (TRPS, qNANO instrument, Izon Science) or Nanoparticle Tracking Analysis (NTA, NanoSight NS300, Malvern Panalytical). The method utilized for each data set is detailed in the figure legends and details reported in Supplementary Materials and Methods.

### 2.5 Protein EV cargo profiling

EV proteins were extracted and analyzed by western blotting as previously reported (Choi et al., 2020) with few modifications as detailed in Supplementary Materials. EV surface marker analysis was performed by MACSPlex Exosome kit (Miltenyi Biotech; no. 130‐108‐813) following the manufacturer's protocol tube for overnight capture on tubes. HER2 protein detection on EV was performed by staining samples with HER2/ErbB2 (29D8)—PE‐conjugated antibody or Isotype Control (Cell Signaling Technology) and CellMask plasma membrane stains (C10046, Life Technologies). HER2‐positive EV were identified on an Amnis ImageStream X MkII (Luminex).

### 2.6 ddPCR assay

ddPCR assays performed on cfDNA and EV‐RNA were from Bio‐Rad Laboratories Inc (*ERBB2*: dHsaCP1000116; dHsaCPE5037554, EIF2C1: dHsaCP2500349, EEF2: dHsaCPE5050049) and PCR reactions were run on T1000 thermal cycler (Bio‐Rad, Hercules, CA, USA). Analysis of ddPCR data was analyzed using QuantaSoft Software, version 1.7 (Bio‐Rad Laboratories, Inc.). EV‐RNA isolation was performed as detailed in Supplementary Materials and Methods.

### 2.7 RNASeq on EV‐RNA

EV‐RNAs were processed with SMART‐Seq® Stranded Kit (Takara Bio USA, Inc.) and libraries were sequenced on the Illumina HiSeq2500 platform by the Next Generation Sequencing Facility at the University of Trento (Italy). Reads were aligned against the human genome (hg38) using STAR (Dobin et al., 2013). Breast cancer tissue data (Ciriello et al., 2015) were downloaded from CBioPortal (https://www.cbioportal.org/), including immunohistochemistry evaluation of HER2.

### 2.8 DNA whole‐exome sequencing

Libraries were prepared with SeqCap EZ HyperCap Workflow version 2.3 (Roche). We utilized 20–50 ng of cfDNA and 100 ng of matched germline DNA (gDNA) that was sonicated to reduce the fragment size to 180–220 bp (Covaris M220). Libraries were sequenced on the Illumina HiSeq2500 platform by the Next Generation Sequencing Facility at the University of Trento (Italy). CNVkit (Talevich et al., 2016) was used for copy number aberration (CNA) detection via segmentation, and Log2 values of cfDNA over control were corrected for purity and ploidy using ClonetV2 (Prandi and Demichelis, 2019). Extended procedure and details are presented in Supplementary Materials and Methods.

### 2.9 HER2 positivity classification of BrCa study patients

We used ddPCR data for all liquid biopsy samples (the highest DNA and RNA ddPCR values of healthy donor samples were used as lower thresholds for DNA and RNA, respectively) and sequencing data for the TCGA tissue samples (DNA amplification threshold was set at 2.6 copies; RNA levels for samples without DNA amplification were considered and the 75% percentile of the distribution was set as lower threshold). For each sample, we then considered the following classes of HER2+ positivity; *Only RNA*: RNA but not DNA signal is higher than the corresponding threshold; *Only DNA*: DNA but not RNA data is higher than the corresponding threshold; *Combo AND*: both DNA and RNA data are above the corresponding thresholds; *Combo OR*: any of DNA or RNA data is higher than the corresponding threshold. The classification performance was estimated based on precision, recall and F1 score as described in Supplementary Materials and Methods.

### 2.10 Statistical analysis

Statistical analysis was performed using GraphPad Prism version 8.0 software (www.graphpad.com) and R software (www.R‐project.org). One‐way ANOVA or t‐test were used as appropriate. Data visualizations were performed using the packages ggplot2 (https://ggplot2.tidyverse.org) and complexHeatmap.

Additional methods and procedures are described on Supplementary Materials and Methods.

## 3 RESULTS

### 3.1 ONCE combines EV isolation with cfDNA extraction from a single plasma aliquot

**FIGURE 1:**
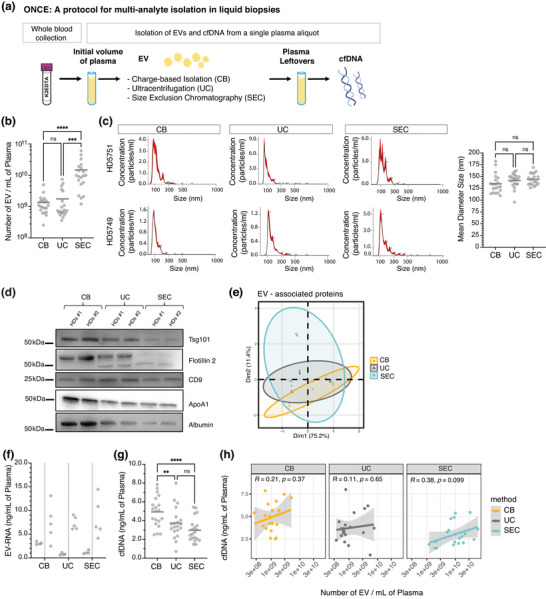
ONCE combines diverse EV isolation methods and cfDNA extraction from a single plasma aliquot. (a) Schematic of the ONCE (ONe Aliquot for Circulating Elements) protocol. Starting from a single plasma aliquot, we propose the ONCE protocol as an approach for sequentially isolating multiple analytes circulating in the blood, here EV and cfDNA. Three diverse EV isolation methods were tested, CB, UC, and SEC. After EV isolation, plasma leftovers are utilized for cfDNA extraction. (b) Quantification of EV by Nanoparticle Tracking Analysis (NTA) measurements. EV were isolated from a cohort of HDs (*n* = 20). Three plasma aliquots were collected from each HDs, and EV were isolated from each aliquot using one of the three specific methods, CB, UC, or SEC. Compared to CB and UC, the SEC method resulted in higher nanoparticle recovery. All plasma for EV isolation was derived from blood collected into K2EDTA tubes. ns: not significant differences; *** *p* = 0.0001, **** *p* < 0.0001 by two‐way ANOVA. (c) Representative profiles of EV samples quantitated in panel b. EV were isolated through ONCE using one of the three specific methods: CB, UC, and SEC. NTA quantitation (NanoSight NTA software v3.2) of EV mean diameter size is compatible with small EV. NTA profiles are from EV of *n* = 2 representative HDs (HD5751 and HD5749) and are shown as a merge of *n* = 5 acquired measurements (videos). n.s.: not significant differences by one–way Anova. (d) Western Blot assay showing the EV‐enriched proteins Tsg101, Flotillin 2, CD9, and the contaminant proteins Apolipoprotein A1 (ApoA1) and Albumin in representative *n* = 2 pool of EV samples from HDs. Plasma from the same individuals was divided into equal aliquots, and EV were isolated from plasma by CB, UC, or SEC. (e) Principal component analysis (PCA) on signals derived from 37 exosomal surface epitopes detected on EV samples of *n* = 3 HDs (technical replicates *n* = 3). EV were isolated by CB, UC, or SEC; EV surface markers were profiled by multiplex bead‐based flow cytometry assay (MACSPlex exosomal kit; Miltenyi Biotec). Each ellipse groups the exosomal epitopes’ expression profile of EVs isolated using one specific method. EV isolated from the same individuals by diverse methods show partial overlap. (f) Quantification of EV‐RNA extracted from EV after isolation. EV‐RNA was quantitated by Agilent RNA pico assay (left) or small RNA assay Kit (right). EV‐RNA samples were derived from *n* = 9 HDs. The plasma of each HDs was divided into three aliquots, and each plasma aliquot was processed for EV isolation by using a dedicated method (CB, UC, SEC). (g) Quantification of cfDNA by Qubit dsDNA HS Assay (Thermo Fisher Scientific). Data are from *n* = 20 HDs. cfDNA was extracted from plasma leftovers collected after EV isolation with a dedicated method (CB, UC, SEC) as in panel a. (h) Scatter plots correlating the amount of recovered cfDNA and EV from each HDs (*n* = 20) across the three EV isolation methods (CB, UC, SEC). No significant positive or negative correlation is derived. *R* and *p*‐values are obtained from a linear model (lm function in R). cfDNA, cell free DNA; EV, extracellular vesicles; EV‐RNA, RNA extracted from EV; HDs, healthy donors; CB, charge—based isolation method; UC, ultracentrifugation; SEC, size—exclusion chromatography. See also associated Figures S1 and S2.

Previously reported procedures for isolating cfDNA and EV require different aliquots of body fluids to purify each circulating component (Diefenbach et al., 2018; Ku et al., 2019). We hypothesized that the concomitant isolation of two diverse analytes (cfDNA and EV) from the same aliquot of a body fluid might be feasible, reduce the amount of valuable blood required for testing, and inherently avoid inter‐sample variability that might affect the sensitivity in detecting relevant cancer information. To this purpose, we developed and tested the ONCE approach as a novel combination of serial procedures for isolating EV and cfDNA from a single plasma aliquot (workflow in Figure 1a). To assess its applicability with a range of isolation methods that enrich for EV, we tested multiple approaches. Specifically, we collected whole blood from a cohort of HDs (*n* = 20) into K2EDTA tubes and separated the plasma fraction. We then divided the collected plasma into three identical aliquots and processed each one of them with a diverse EV isolation method: (1) a charge–based (CB) method (Liangsupree et al., 2021; Notarangelo et al., 2019), previously used for analyses on EV isolated from cancer patient plasma (Pasetto et al., 2021); (2) ultracentrifugation (UC), the most commonly utilized EV isolation method (Thery et al., 2006); (3) size‐exclusion chromatography (SEC) on qEV/70 nm columns associated with an automatic fraction collector system certified as a medical device (IZON Science LTD) (Gardiner et al., 2016).

After EV enrichment, we recovered the plasma leftovers and processed them for cfDNA extraction through a silica‐based membrane technology (Diefenbach et al., 2018; Lee et al., 2018) (Figure 1a).

First, we compared the efficiency of the three considered methods for EV isolation by performing nanoparticle tracking analysis (NTA) measurements. While the SEC method resulted in the highest nanoparticle recovery efficiency (Figure 1b), the profiles and the mean diameter of the EV‐enriched samples were similar across the methods, thereby suggesting that all three methods allow efficient isolation of small EV (Figure 1c).

Immunoblot analysis detected protein markers commonly associated with exosomes (Tsg101, Flotillin‐2, CD9) as well as commonly co‐isolated contaminants such as Apolipoprotein A1 and Albumin in line with previously reported heterogeneous mixtures of vesicular (small EV) and non‐vesicular (i.e. lipoproteins) nanoparticles (Figure 1d) (Holcar et al., 2021), with distinctive expression profiles across diverse isolation methods. To confirm the distinctiveness of EV obtained by CB, UC or SEC, we compared the detection levels of a panel of 37 exosome surface epitopes by MACSPlex immunocapture assay (Ekstrom et al., 2022). PCA analysis showed partial overlap among the samples isolated with the different methods suggesting that the expression levels of the analyzed epitopes are only partially concordant through diverse methods (Figure 1e).

We then evaluated the recovery of nucleic acids associated with EV isolated from HDs using the three diverse methods. Starting from small EV isolated from an aliquot of plasma (1.8 mL), we extracted a minimal amount of DNA (less than 1 ng of DNA per mL of plasma) across the three EV isolation methods (Figure S1a), thereby confirming previous data reporting DNA mainly carried by large EV (Vagner et al., 2018). By using the same volume of plasma (1.8 mL) for EV isolation by CB, UC, or SEC, we were able to extract a good amount of RNA (to 10–15 ng of RNA per mL of plasma) (Figure 1f) with comparable fragment size length across the tested EV isolation methods (Figure S1b).

Once we assessed the differences in recovered EV‐RNA among CB, UC, and SEC, we analyzed the relative plasma leftovers (EV‐depleted plasmas) for the subsequent extraction of cfDNA. EV‐depleted plasmas obtained after the CB‐mediated EV isolation method resulted in the highest cfDNA recovery when compared to EV‐depleted plasmas collected after UC and SEC processing (Figure 1g); in contrast, the size length of the extracted cfDNA fragments was comparable among the three (Figure S1c). Thus, the CB method resulted as the most convenient approach for combining the extraction of cfDNA with the isolation of EV. Also, we assessed that the efficiency of cfDNA recovery was not directly related to EV extraction's efficiency. We did not observe a significant correlation between the amount of recovered cfDNA and the number of isolated EV across the three methods for EV isolation (CB, UC, and SEC), thus suggesting that the differences in cfDNA recovery between the three methods are independent of the efficiency of EV isolation (Figure 1h).

To further assess whether the efficiency of cfDNA extraction from EV‐depleted plasma was affected by the prior isolation of EV, we checked if the levels of cfDNA extracted by performing ONCE with the CB isolation method were comparable with the cfDNA levels obtained without any plasma processing steps for the EV isolation (I CONTROL and II CONTROL) (Figure S2a). To this purpose, we collected blood from *n* = 20 HDs. Two types of tubes, K2EDTA and BCT Streck tubes, were used. Each volume of the separated plasma sample was split into three aliquots (1.8 mL/each). The three aliquots of plasma were then processed, each with one of the three following protocols (Figure S2a):

- ONCE Protocol, the aliquot is diluted in PBS1X and incubated with EV‐capture beads for EV isolation; the diluted EV‐depleted plasma leftovers are then utilized for cfDNA extraction.
- I CONTROL protocol, the aliquot is processed to purify cfDNA according to the protocol commonly utilized for cfDNA isolation (Chen et al., 2020).
- II CONTROL protocol, the aliquot is first diluted in PBS1X (as in i) and then processed to purify cfDNA, as in ii, to check whether the sole dilution of the plasma (performed to reduce plasma viscosity) interferes with the efficiency of cfDNA recovery.

By comparing the ONCE protocol with controls (I CONTROL and II CONTROL protocols), no significant differences were detected in the amount or in the size length of cfDNA recovered, regardless of the blood tube type and the relevant associated preservatives (Figure S2b–g).

Altogether, our data indicate that the CB method for EV isolation within the framework of the ONCE approach allows for the combined isolation of cfDNA and EV and ensures the preservation of the isolated DNA from a single plasma aliquot.

In light of these results, for the subsequent experiments, we opted for the CB‐mediated EV isolation method in the framework of ONCE as this method combines an EV recovery (in terms of the EV number isolated per mL of plasma) comparable to UC with an optimal cfDNA yield, higher speed and easier scalability.

### 3.2 Processing of liquid biopsies from early‐stage BrCa patients for serial isolation of EV and cfDNA

**FIGURE 2:**
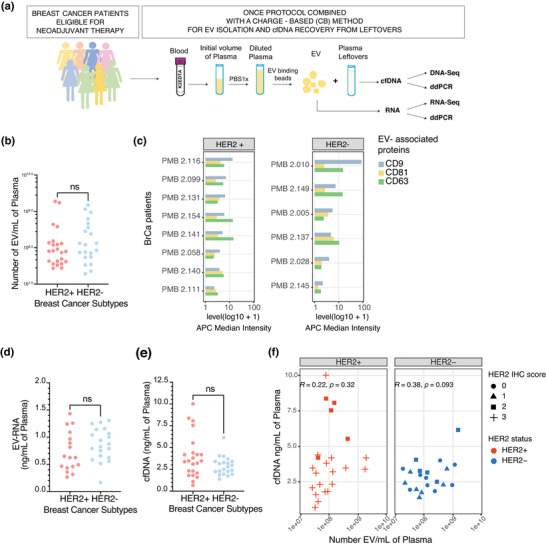
EV and cfDNA are concomitantly and efficiently extracted from early‐stage BrCa liquid biopsies. (a) Schematic of the application of the ONCE protocol for the liquid biopsy multi‐analyte characterization of BrCa patients. An aliquot of whole blood was collected from *n* = 44 early‐stage non metastatic BrCa patients into K2EDTA tubes, and the separated plasma (1.8 mL) was processed by ONCE with the CB isolation method. EV and cfDNA are sources of circulating nucleic acids for investigating tumor biomarkers by RNA‐Seq/DNA‐Seq and /or ultra‐sensitive ddPCR. The dilution of plasma with PBS1x is an essential step to reduce plasma viscosity, thereby facilitating the binding of the beads to the EV and guaranteeing the performance of the CB method. (b) Quantification by TRPS (Tunable Resistive Pulse Sensing) measurements of the EV isolated from the (*n* = 44) BrCa patients stratified by HER2+ and HER2‐ subtype, based on histopathological classification. n.s.: not significant differences by *t*‐test. (c) Detection of CD8, CD63, and CD81 tetraspanins by MACSPlex exosome assay on EV isolated by CB method throughout ONCE protocol. *X* axis plots the detection signal (Median Intensity level on APC channel) in log10+1 scale. *Y* axis reports BrCa patient code. Data are from *n* = 8 HER2+ and *n* = 6 HER2‐ BrCa patients. (d) Quantification of EV‐RNA extracted from EV after isolation by CB method. EV‐RNA was quantitated by Agilent RNA pico assay. EV‐RNA samples were derived from *n* = 17 HER2+ and *n* = 20 HER2‐ BrCa patients. n.s.: not significant differences by *t*‐test. (e) Quantification of cfDNA by Qubit dsDNA HS Assay (Thermo Fisher Scientific). cfDNA was extracted from plasma leftovers, after EV enrichment, according with ONCE protocol. Data are from *n* = 23 HER2+ and *n* = 21 HER2‐ early‐stage BrCa. n.s.: not significant differences by one–way Anova. (f) Scatter plot of levels of cfDNA (ng/mL of plasma) and EV (number of EV/ mL of plasma) recovered by ONCE from *n* = 44 BrCa patients. Data are stratified by HER2 status (HER2+ vs. HER2‐). The IHC score, derived from tissue biopsies, is shown with symbols. CfDNA, cell free DNA; EV, extracellular vesicles; EV‐RNA, RNA extracted from EV; BrCa, breast cancer; HER2+, HER2 positive; HER2, HER2 negative; IHC, immunohistochemistry. See also Figure S3.

To assess the potential of detecting cancer‐derived cargo in liquid biopsies from cancer patients, we tested ONCE in a cohort of early‐stage BrCa patients (*n* = 44) prospectively enrolled between 2017 and 2019 at the Santa Chiara hospital in Trento (Italy) and subjected to neoadjuvant chemotherapy (NAC) (Table 1). Specifically, we sought to test the ability to assess HER2 status in patients’ circulation by querying multiple analytes. To this end, baseline plasmas from single aliquots of blood collected into a K2EDTA tube were utilized to extract cfDNA after EV isolation by the CB method (Figure 2a).

**TABLE 1:** Demographic table of breast cancer patients’ study cohort.

Characteristic	Patients (*N* = 56)
Age at baseline	
Median age (range)—yr	52.9 (28–77)
40 years—no. of patients (%)	4 (7.1%)
>40 years—no. of patients (%)	52 (92.8%)
Subtype classification—no. of patients (%)	
HER2 positive: HER2+	17 (30.3%)
Luminal B (HER2+): ER+ a/o PR+ HER2+	10 (17.8%)
Triple Negative: ER‐ PR‐ HER2‐	16 (28.5%)
Luminal B (HER2‐): ER+ a/o PR+ HER2‐	13 (23.2%)
Histological subtype—no. of patients (%)	
Ductal	51 (91.0%)
Lobular	2 (3.6%)
Infiltrating NST	3 (5.4%)
HER2 expression status (IHC; score)—no. of patients (%)	
0, 1+ (negative)	17 (30.3%)
2+ (ambiguous)	12 (21.4%)
3+ (positive)	26 (46.4%)
HER2 amplification status (FISH)^*^—no. of patients (%)	
Positive	9 (16.0%)
Negative	7 (12.5%)
Not performed	40 (71.4%)
Metastasis (M; pathological stage)—no. of patients (%)	
M0	56 (100%)
Clinical cancer stage—no. of patients (%)	
Stage IA‐IB	6 (10.7%)
Stage IIA	19 (33.9%)
Stage IIB	16 (28.6%)
Stage IIIA	6 (10.7%)
Stage IIIB	3 (5.3%)
Stage IIIC	3 (5.3%)
n.a.	3 (5.3%)

* According to ASCO‐CAP guidelines (Wolff AC, Hammond MEH, Allison KH, Harvey BE, Mangu PB, Bartlett JMS, et al. Human Epidermal Growth Factor Receptor 2 Testing in Breast Cancer: American Society of Clinical Oncology/College of American Patholo‐ gists Clinical Practice Guideline Focused Update. J Clin Oncol 2018;36(20):2105‐22 doi 10.1200/JCO.2018.77.8738).

We first quantified the isolated EV by tunable resistive pulse sensing (TRPS) technology. We observed no significant differences in EV concentration between HER2+ and HER2‐ subtypes (Figure 2b) (mean range across subtypes: 2.40E+08 and 3.10E+08 EV/mL of plasma, respectively). By western blot analysis (Figure S3a) and MACSPlex immunocapture assay (Figure 2c), we confirmed the expression of EV‐enriched proteins, including Tsg101, Flotillin 1/2, and tetraspanins (CD9, CD63, CD81) at similar levels among a subset of the HER2+ and HER2‐ samples. We also confirmed the heterogeneity of the isolated EV‐enriched samples by detecting makers of co‐isolated non‐vesicular nanoparticles such as Albumin and LipoproteinA1 (Figure S3a).

Next, to evaluate the EV‐associated nucleic acid cargo, we extracted DNA and RNA from these early‐stage BrCa patients’ EV (EV‐DNA and EV‐RNA). A minimal amount of EV‐DNA (mean: 0.0140 ng of DNA/ mL of plasma) was obtained from patient plasma (Figure S3b). In contrast, higher DNA recovery was obtained from synthetic liposomes containing artificial DNA and from EV of metastatic BrCa patient plasmas, here included as internal controls (Figure S3b–d). Regarding the EV‐RNA, we consistently extracted comparable amounts among HER2+ (mean: 0.729 ng/mL of plasma) and HER2‐ samples (mean:0.866 ng/mL of plasma) (Figure 2d and Figure S3e).

Finally, we processed the EV‐depleted plasma, collected after EV isolation, for cfDNA extraction as previously described (Figures 1a and 2a). The concentration of cfDNA was comparable in the two BrCa subtypes (mean HER2+: 3.9 ng/mL; mean HER2‐:2.9 ng/mL) within the early‐stage cohort (Figure 2e and Figure S3f).

Furthermore, in the same BrCa patient cohort, no correlation was observed between the amounts of cfDNA and EV, regardless of the HER2 status obtained on tissue biopsies according to clinical classification (Figure 2f).

Altogether our data suggested that HER2+ and HER2‐ patients exhibit comparable quantity and quality of EV and cfDNA, thereby supporting the opportunity of profiling both analytes for cancer‐associated biomarkers.

### 3.3 cfDNA and EV represent circulating components that reflect the patient tumor molecular features assessed on tissue biopsies

We sought to provide proof of principle validation of ONCE–derived analytes for cancer cargo detection in liquid biopsies by taking advantage of the possibility of jointly screening cfDNA with EV cargo. To detect molecular alterations associated with BrCa, we performed DNA whole‐exome sequencing (WES) (mean coverage = 537x, min = 282x, max = 731x) and RNA deep sequencing (mean depth = 88 M reads, min 56 M reads, max = 97 M reads, detecting 13009 transcripts on average at cpm > 5, min = 8545, max = 19113) respectively on cfDNA and EV‐RNA isolated from two HER2+ (PMB2.8 and PMB2.36) and two HER2‐ (PMB2.26 and PMB2.30) BrCa patients (Table S1 for patient marker status, Table S2 for WES coverage, Table S3 for cfDNA and EV quantification and Table S4 for RNAseq statistics).

**FIGURE 3:**
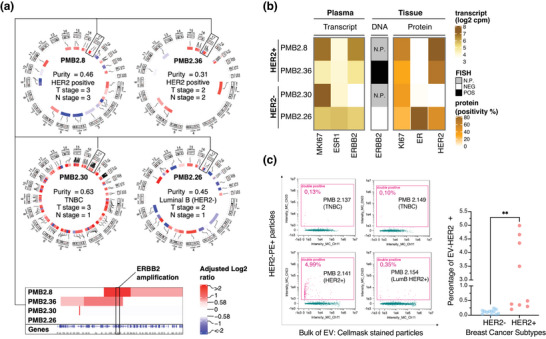
Circulating cfDNA and EV in early stage BrCa patients are a remarkable source for detecting genetic and molecular alterations. (a) Circos plots of cfDNA analyzed by whole‐exome DNA‐Seq (WES) of *n* = 4 representative BrCa patients. Squares outline the *ERBB2* gene locus (Chr.17q12) shown as linear visualization in the inset; *ERBB2* gene amplification is detectable in the cfDNA of HER2+ BrCa patients’ plasma (PMB2.8 and PMB2.36), and not in the cfDNA of HER2‐ BrCa patients (PMB 2.30 and PMB2.26). Purity indicates the circulating tumor content. (b) Heatmap showing the detection of relevant breast cancer biomarkers in *n* = 4 patients: PMB2.8 and PMB2.36 (HER2+), PMB2.30 and PMB2.26 (HER2‐). Transcripts were detected by RNA‐Seq from EV‐RNA from liquid biopsies. Proteins were assessed by IHC on tissue biopsies. DNA amplification of *ERBB2* gene was assessed by FISH on tissue biopsies. *ERBB2* (HER2), ESR1 (Estrogen receptor alpha) and MKI67 (Marker of proliferation Ki‐67). (c) Representative plots and quantification of HER2+ EV subpopulations by imaging flow cytometry (Amnis Imagestream^x^ MK II). Plots show CellMask staining utilized to visualize the bulk of lipid nanoparticles on *x*‐axis (Intensity_MC_Ch_11) and PE‐HER2 Antibody staining on y‐axis (Intensity_MC_Ch_03). Data are from technical replicates of measurements performed on EV samples isolated from *n* = 3 HER2‐ and *n* = 3 HER2+ BrCa patients (PMB). ** *p* = 0.0041 by *t*‐test. cfDNA, cell free DNA; EV, extracellular vesicles; EV‐RNA, RNA extracted from EV; BrCa, breast cancer; HER2+, HER2 positive; HER2‐, HER2 negative; IHC, immunohistochemistry. See also Figure S4.

As illustrated by the circos plots (Figure 3a), DNA‐Seq of the cfDNA samples detected several cancer‐related genomic alterations and confirmed the amplification of *ERBB2* (on chromosome 17) of patients PMB 2.8 and PMB 2.36, in line with diagnostic tissue biopsy in situ assessment (Table S1). Multiple cancer–related genomic alterations, but no *ERBB2* amplification, were present in the cfDNA samples from patients PMB2.30 and PMB2.26, classified as HER2‐ (Figure 3a). RNA‐Seq of the EV‐RNA from the same patients showed that transcripts encoding for HER2 were detectable on samples PMB2.8 and PMB2.36, both HER2+ (Figure 3b), but not on the sample PMB2.30 (HER2‐). Interestingly, the sample PMB2.26 was classified as HER2‐ based on the absence of *ERBB2* genomic amplification by FISH assay. However, the corresponding IHC on the tissue biopsy showed low to moderate membrane immunoreactivity to HER2 antibody in 50% of tumor cells (Table S1), compatible with the EV‐RNA sequencing data, and overall suggesting a potential HER2 overexpression regulation at the transcriptional or post‐transcriptional level (Daemen & Manning, 2018; Kraus et al., 1987; Li et al., 2020; Liu et al., 2018; Wang et al., 2017). Additionally, transcripts encoding for markers of prognostic value in BrCa, such as KI67 (a marker of proliferation Ki‐67) and estrogen receptor alpha (ER) (Duffy et al., 2017), were differentially detected in both HER2+ (PMB2.8 and PMB2.36) and HER2‐ samples (PMB2.30 and PMB2.26) (Figure 3b; Plasma) in line with tissue biopsy data (Figure 3b; Tissue/Protein). Altogether, the sequencing data, despite the limited sample size, confirmed cfDNA obtained through ONCE as a circulating source of information for cancer profiling and highlighted the relevance of the EV‐RNA cargo as a powerful additional component for cancer biomarkers detection in multi‐analyte liquid biopsies. Additionally, by western blotting analysis on EV‐associated proteins released in the medium by HER2+ (SKBR3) and HER2‐ (MDAMB231) breast cancer cell lines, we assessed EV as carriers of the HER2 biomarker in the medium from HER2+ cancer cell line only (Figure S4a). By using immuno‐mediated staining in combination with imaging flow cytometry, we were able to confirm the association of HER2 to the surface of EV subpopulations and their differential abundance among a subset of *n* = 3 BrCa HER2+ and *n* = 3 HER2‐ samples (Figure 3c and Figure S4b for IgG isotype control). In conclusion, in this proof‐of‐principle experiments, we assessed that ONCE‐derived cfDNA and EV represent an informative source for investigating cancer biomarkers, such as HER2, in the circulation, as genomic and molecular features are representative of matched tissue‐based investigations.

### 3.4 Integrating EV‐RNA and cfDNA data increases precision in identifying the HER2 status at an early stage of BrCa

After demonstrating that cfDNA and EV can be isolated from BrCa patients with no significant differences in the recovered amount across the two subtypes (Figure 2b and e) and that they allow the detection of cancer–associated alterations (Figure 3a,b), we sought to quantify the ability of such circulating nucleic acids to screen for the status of a critical BrCa biomarker, such as HER2 in early stage BrCa liquid biopsies. We hypothesized that the combination of both EV‐RNA and cfDNA‐based quantitative assessments would increase the detection performance by overcoming potential limitations on a patient basis (e.g. a low fraction of circulating tumor DNA, a low proportion of cancer‐related EV in the circulation).

**FIGURE 4:**
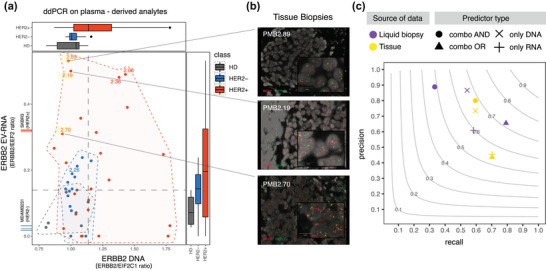
Integrating data from circulating cfDNA and EV‐RNA strengthens the potential of liquid biopsies in early‐stage BrCa. (a) Concomitant ddPCR of *ERBB2* gene in cfDNA and EV‐RNA transcript. Red dots: HER2+ BrCa (*n* = 24); blue dots: HER2‐ BrCa (*n* = 14) (stratification based on tissue biopsies data); grey dots: HDs (*n* = 7). Marginal boxplots show distributions for each group of samples. Colored areas contain all data points of each specific class (HDs, HER2+, or HER2‐). Grey lines mark the thresholds based on HDs distributions for HER2 positivity prediction (maximum level of RNA and DNA detected in HDs is set as threshold). Measurements from EV‐RNA of SKBR3 (HER2+) and MDAMB231 (HER2‐) breast cancer cell lines (*n* = 3 replicates) are indicated on the *Y* axis by red and blue lines, respectively. Three labeled BrCa patients are included in panel b. (b) Fluorescent in situ hybridization (FISH) pictures of matched BrCa tissue biopsies show HER2 gene amplification. Quantification of gene amplification based on HER2 (red) and CEP17 (green) ratios according to the American Society of Clinical Oncology (ASCO)/College of American Pathologists (CAP) guidelines indicates HER2/CEP17 ratios of 3.0 (PMB2.89), 5.8 (PMB2.70), and 2.9 (PMB2.19). (c) Performance of HER2 status classifiers: combining cfDNA and EV‐RNA data increases the performance of liquid biopsy biomarker detection. The liquid biopsy classifier (violet) is based on panel a data. The tissue biopsies classifier (yellow) uses data from Figure S5. Symbols indicate which analyte is included in the classifiers (only DNA, RNA, or both). Samples with values higher than the thresholds are classified as HER2+ based on one of the following rules: only RNA: only the signal of RNA reaches the threshold; only DNA: only the signal of DNA reaches the threshold; Combo AND: both DNA and RNA signals reach the threshold; Combo OR: signal for any DNA or RNA reaches the threshold. Precision is the ratio between predicted true positives and the total number of predicted positives. The recall is the ratio between predicted true positives and the total number of positives in the population. Lines in the background show the F1 score (harmonic mean between recall and precision). The F1 score is a measurement performance score ranging from 0 (lowest recall; lowest precision) to 1 (highest recall; highest precision). cfDNA, cell‐free DNA; EV, extracellular vesicles; EV‐RNA, RNA extracted from EV; ddPCR, droplet digital PCR; HDs, healthy donors; BrCa, breast cancer; HER2+, HER2 positive; HER2‐, HER2 negative; HDs, healthy donors. See also Figure S5.

We analyzed EV‐RNA and cfDNA by ddPCR, a fast technique widely applied to screen large numbers of samples that has already been successfully used in clinical liquid biopsies‐based analyses for its high sensitivity and specificity (Olmedillas‐Lopez et al., 2022; Silveira et al., 2021). To ensure a large enough set of BrCa patient plasmas with EV‐RNA and cfDNA yields adequate to run the two ddPCR assays, we extended the study cohort to additional patients from the same prospectively collected clinical cohort (Table 1). Thus, we characterized a set of *n* = 38 BrCa (HER2+ and HER2‐) samples for *ERBB2* gene amplification on cfDNA and *ERBB2* transcript expression on EV‐RNA. Amplification on cfDNA was determined as the ratio between circulating *ERBB2* and reference gene *EIF2C1* by using a previously reported ddPCR assay for *ERBB2* copy number alteration on BrCa tissues (Tantiwetrueangdet et al., 2018). The expression of EV‐RNA was calculated as a ratio between the detection of fragments corresponding to ERBB2 and the commonly utilized tissue reference gene *EEF2* (Ersahin et al., 2014). To evaluate the specificity of this ddPCR assay, we included a set of *n* = 7 HDs (to define the thresholds on *ERBB2* amplification and overexpression) and EV‐RNA extracted from conditioned cultured medium of HER2+ (SKBR3) and HER2‐ (MDAMB231) breast cancer cell lines. ddPCR data showed that 57% of the HER2‐ BrCa patients (Figure 4a, blue dots) group with the HDs, while 83% of HER2+ BrCa patients (Figure 4a, red dots) show higher signals than HDs on *ERBB2* on EV‐RNA (*ERBB2/EEF2*; Figure 4a, *y*‐axis) and cfDNA (*ERBB2/EIF2C1;* Figure 4a, *x*‐axis). HER2‐ patients never show cfDNA *ERBB2* gene amplification, whereas 8 of them show mild transcript overexpression in the lowest range of expression of HER2+ patients. ddPCR data on EV‐RNA and cfDNA showed concordant marked positivity for about 30% of the HER2+ BrCa samples. In comparison, 25% (*n* = 6 out of 24 red dots) and about 20% (*n* = 5 out of 24 red dots) of HER2+ samples were correctly classified by EV‐RNA only and cfDNA only, respectively. EV‐RNA resulted of particular importance to capture samples that were classified positive for HER2 based on *ERBB2* gene amplification by FISH on tissue biopsies (Figure 4b), but for which the amplification was not detectable in cfDNA (as PMB2.19, PMB2.70, PMB2.89).

To estimate the relevance of combining EV‐RNA and cfDNA signal in BrCa, we compared the classification performance of every single analyte (“*only DNA*”, “*only RNA*”) versus combined analytes (“*combo OR*”, “*combo AND*”, meaning that high level of either one or both analytes is required for positivity; see Methods) by focusing on the presence or absence of *ERBB2* amplification or HER2 overexpression (see Method section HER2 positivity classification of plasma and tissue samples). The prediction accuracy was measured by the F1 score (Figure 4c, grey lines), that is the harmonic mean between the recall (the fraction of HER2+ cases that can be detected; Figure 4c, *x*‐axis) and the precision (the fraction of cases that are correctly classified as HER2+; Figure 4c, *y‐*axis); the higher the F1 score, the better the classification accuracy. To compare liquid biopsy‐based results and tissue‐based results, we similarly quantified the classification performance from DNA and/or RNA sequencing data from the TCGA BrCa patient tissue cohort (*n* = 537) (Tomczak et al., 2015) (Figure S5a).

We observed that the highest performance is obtained by the combination of the two analytes (*combo OR*, violet triangle) and that the results obtained from the liquid biopsy are not inferior to those obtained from tissue (yellow triangle) (Table S5). Thus, using the information of both analytes in liquid biopsy (*combo OR*, violet triangle) leads to classification performance comparable to the *combo AND* approach in tissues (yellow circle), which is the best‐performing predictor for tissue data.

Altogether these data demonstrate that the power of ONCE–derived EV‐RNA and cfDNA analysis for capturing HER2 positive cases is comparable to that obtained by sequencing of tissue nucleic acids.

## 4 DISCUSSION

Integrating multiple circulating analytes information is challenging (Geeurickx & Hendrix, 2020). The proposed ONCE protocol allows the implementation of liquid biopsies‐based tests limiting the blood volume to be collected from patients and concomitantly improving operational and analytical procedures. This is particularly relevant for pediatric and severely debilitated patients, for which accessibility to blood draws may be limited. Processing single aliquots of plasma is less time‐consuming and more cost‐effective as it halves the cost of blood draws and subsequent laboratory processing compared to using multiple aliquots. Furthermore, on the analytical side, using a single aliquot to analyze two components allows discriminating among technical and biological effects, such as the possibility to distinguish if low levels of circulating cfDNA or EV amounts are likely due to degradation issues versus low shedding rate of the tumor cells.

Notably, the high quality of ONCE‐isolated circulating nucleic acids (cfDNA and EV‐RNA) is suitable for ultra‐sensitive techniques such as the ddPCR and NGS and the subsequent potential screening of genetic alterations at early‐stage of cancer.

By proposing the analysis of EV as a circulating component in combination with cfDNA, the ONCE protocol enables the detection of ERBB2/HER2 biomarker in patients where the relevant biomarker signal presents at the transcriptional level would be missed by analysis of cfDNA only. Indeed, we showed that the ONCE protocol might be an informative approach to monitor *ERBB2* in breast cancer patients without genomic *ERBB2* amplification or patients that are HER2 positive at diagnosis and turn negative after multiple cycles of therapy (Bon et al., 2020; Branco et al., 2019).

In this work, we show the applicability and utility of the combined analysis of cfDNA and EV‐RNA for the detection of *ERBB2*, which specifically leverages the fact that not all HER2+ tumors harbor genomic amplifications of the gene (Horimoto et al., 2019). Hence EV‐RNA represents an orthogonal source of information. The same approach may be of limited utility for other biomarkers for which DNA or RNA data alone may suffice.

In addition to the EV‐RNA component, the advantage of including EV as a player in multi‐analyte liquid biopsy approaches is represented by the variety of molecular features embedded into their cargo that allows extending cancer biomarkers analysis to other biomolecules, such as proteins exposed on their surface. Using imaging flow cytometry, we identified subpopulations of EV carrying HER2 and estimated their relative abundance compared to the total number of circulating EV. A further implementation of our approach by combining multiple cancer biomarkers and/or platforms to screen large numbers of samples in a short time (i.e., ELISA assays) would represent a significant advance for investigating EV in liquid biopsies.

Additionally, diverse methods for EV isolation can be adapted to the ONCE protocol and selected based on recommendations proposed by MISEV 2018 (Thery et al., 2018) and by specific research interests on EV as companion analytes for liquid biopsies. Importantly, nanoparticles isolation methods based on biophysical properties, such as UC, SEC, and CB, are recognized to enrich for EV, while co‐isolating a heterogeneous mixture of non‐vesicular nanoparticles, such as lipoproteins (Holcar et al., 2021; Phillips et al., 2021). The heterogeneity of such EV‐enriched samples may represent a source for potential exploration of a variety of similar nanoparticles, including protein complexes associated with RNA and DNA (Driedonks et al., 2020; Juul‐Madsen et al., 2021).

Altogether, the data here presented on the detection of the *ERBB2* status at early‐stage disease offer a model for concomitantly exploring EV and cfDNA from a limited amount of plasma.

Further studies will be essential for the validation on more extensive sample collections, diverse tumor types, and biomarkers for the future implementation of multi‐analyte liquid biopsy‐based assays in the clinical setting.

## AUTHOR CONTRIBUTIONS

**Vera Mugoni**: Conceptualization; Data curation; Formal analysis; Investigation; Methodology; Writing—original draft; Writing—review and editing. **Yari Ciani**: Conceptualization; Data curation; Formal analysis; Investigation; Methodology; Writing—original draft; Writing—review and editing. **Orsetta Quaini**: Data curation; Investigation; Methodology; Writing—review and editing. **Simone Tomasini**: Data curation; Investigation; Methodology; Writing—review and editing. **Michela Notarangelo**: Data curation; Investigation; Methodology; Writing—review and editing. **Federico Vannuccini**: Data curation; Formal analysis; Investigation; Writing—review and editing. **Alessia Marinelli**: Data curation; Formal analysis; Writing—review and editing. **Elena Leonardi**: Data curation; Formal analysis; Writing—review and editing. **Stefano Pontalti**: Data curation; Formal analysis; Investigation; Writing—review and editing. **Angela Martinelli**: Data curation; Investigation; Writing—review and editing. **Daniele Rossetto**: Data curation; Investigation; Writing—review and editing. **Isabella Pesce**: Methodology; Writing—review and editing. **Mattia Barbareschi**: Conceptualization; Data curation; Investigation; Supervision; Validation; Writing—review and editing. **Antonella Ferro**: Data curation; Investigation; Supervision; Validation; Writing—review and editing. **Orazio Caffo**: Data curation; Investigation; Supervision; Validation; Writing—review and editing. **Gerhardt Attard**: Conceptualization; Investigation; Writing—review and editing. **Dolores Di Vizio**: Conceptualization; Investigation; Writing—review and editing. **Caterina Nardella**: Conceptualization; Data curation; Formal analysis; Investigation; Methodology; Project administration; Supervision; Validation; Visualization; Writing—original draft; Writing—review and editing. **Francesca Demichelis**: Conceptualization; Data curation; Formal analysis; Funding acquisition; Investigation; Methodology; Project administration; Resources; Software; Supervision; Validation; Visualization; Writing—original draft; Writing—review and editing.

## CONFLICT OF INTEREST STATEMENT

An Italian Patent Application no. 102021000027854 was filed on October 29th, 2021, on the ONCE methodology (authors involved are VM, YC, OQ, MN, VGD, CN, FD). All remaining authors have declared no conflicts of interest.

## Supporting information

Supporting Inforamtion

Supporting Information

Supporting Information

## Data Availability

The datasets used and/or analyzed during the current study are available from the corresponding author upon request.
