# Supplementary material for: Integrating extracellular vesicle and circulating cell‐free DNA analysis using a single plasma aliquot improves the detection of HER2 positivity in breast cancer patients
Source: J Extracell Biol. 2023 Sep 25;2(9):e108. doi: 10.1002/jex2.108 (PMC10688391; doi:10.1002/jex2.108)
Supplement: Supplementary file 1 — Supporting Inforamtion [file JEX2-2-e108-s002.pdf]

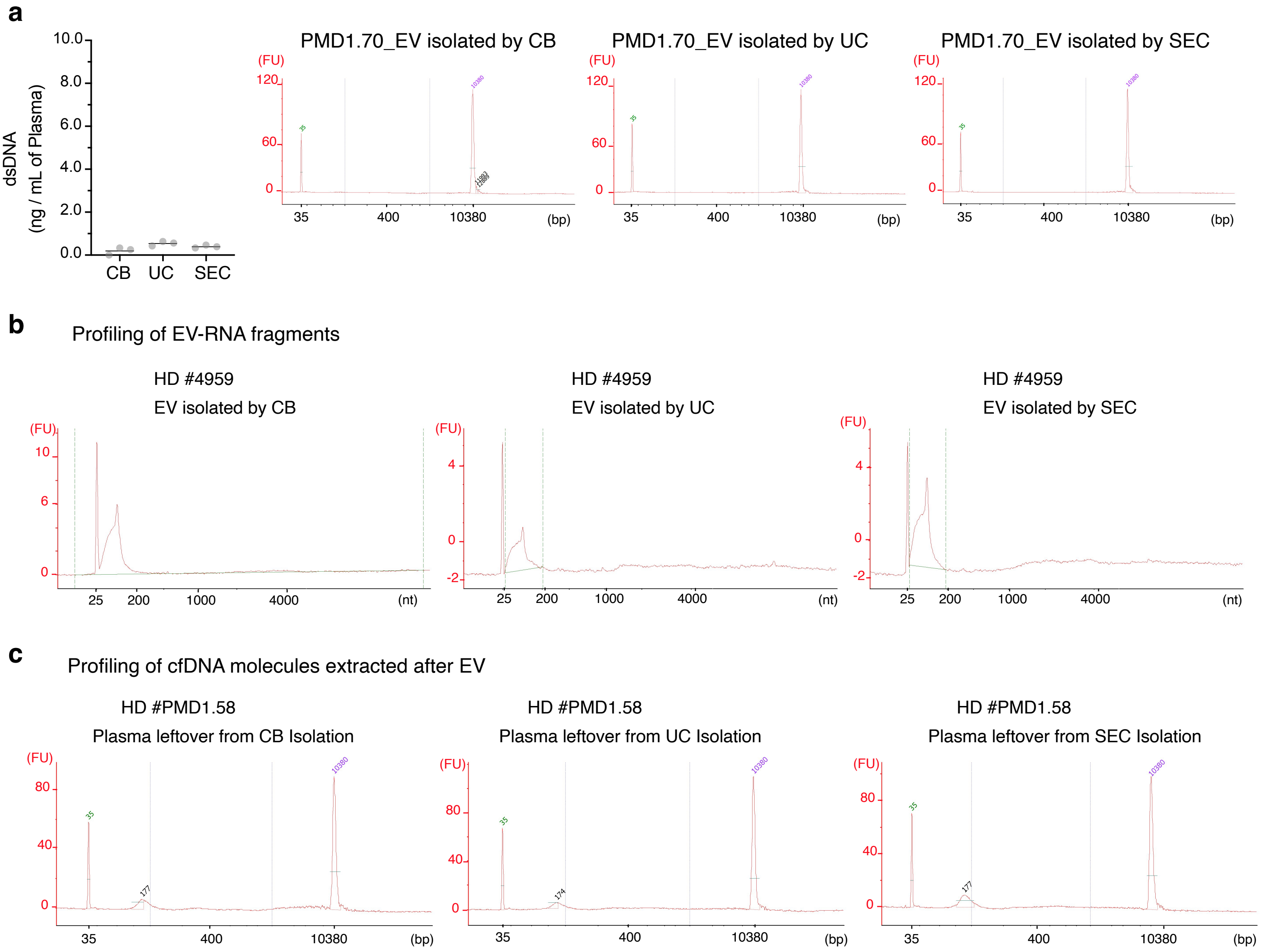

Supplementary Figure 1
Mugoni V., Ciani Y. et al; 2023

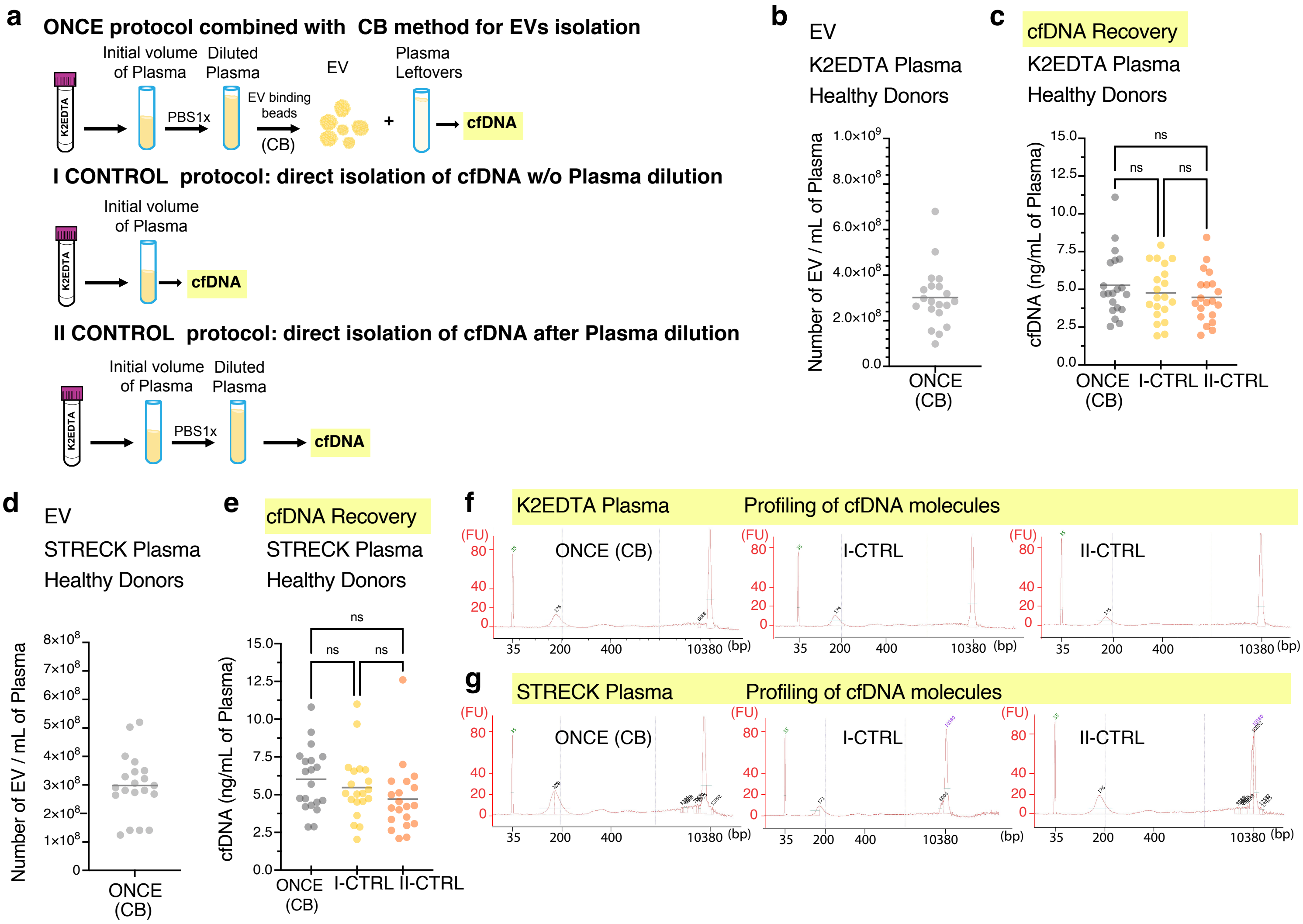

Supplementary Figure 2
Mugoni V., Ciani Y. et al; 2023

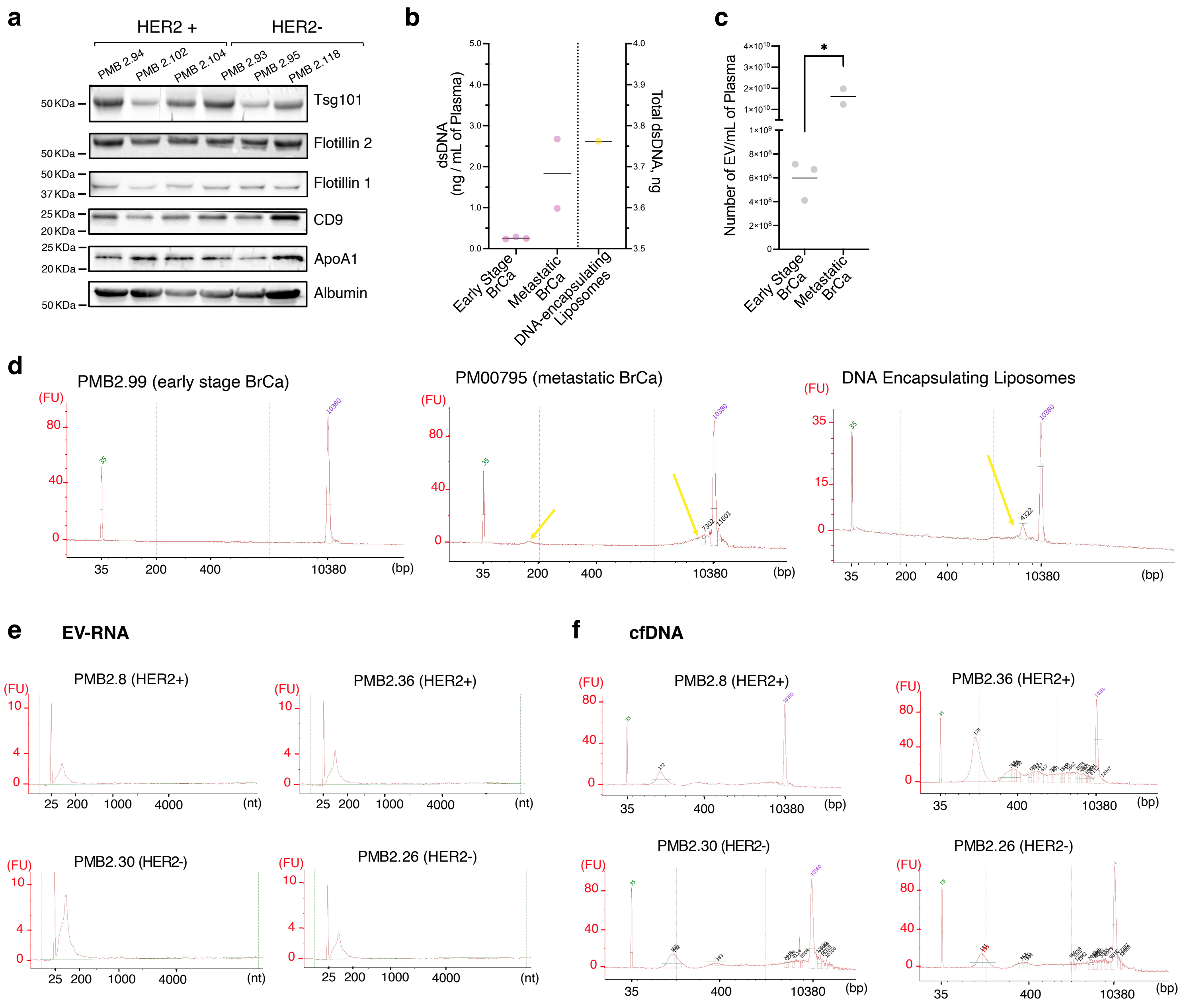

Supplementary Figure 3
Mugoni V., Ciani Y. et al; 2023

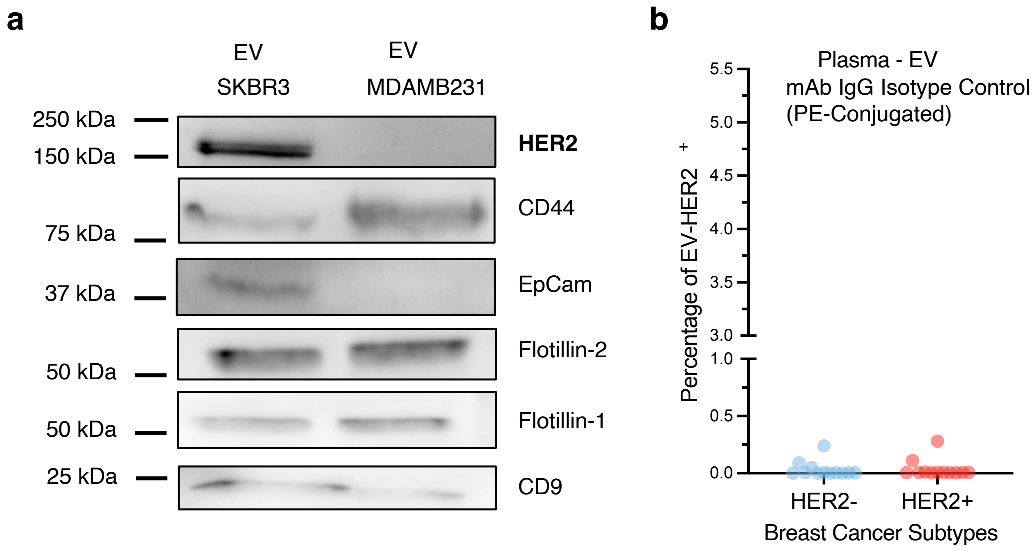

Supplementary Figure 4
Mugoni V. & Ciani Y. et al; 2023

a

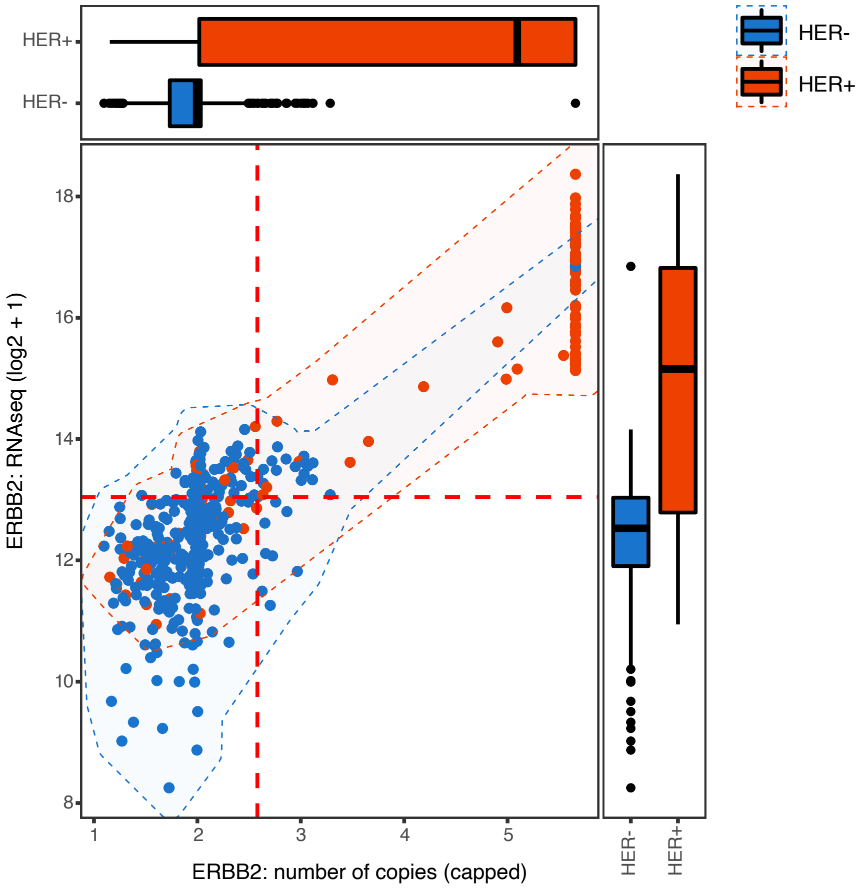

Supplementary Figure 5
Mugoni V., Ciani Y. et al; 2023

Supplementary Figure 1 related to Main Figure 1. Short fragments of nucleic acids are isolated from plasma.

a. Quantification of EV-associated dsDNA and representative bioanalyzer profiles obtained by Agilent HS DNA bioanalyzer assay. The dsDNA was quantitated by Qubit dsDNA HS assay after extraction from EV samples isolated by CB, UC or SEC. EV samples were from plasma (1.8mL) of n=3 HDs. Bioanalyzer plots were from healthy donor PMD1.70.

b. Representative profiles of RNA samples isolated from EV (EV-RNA) obtained by Agilent RNA 6000 Pico bioanalyzer assay. Bioanalyzer internal standard is at 25bp. EV were separated from plasma of HD #4959 by using three diverse methods: CB, UC or SEC.

c. Representative bioanalyzer profiles of cfDNA obtained by Agilent HS DNA bioanalyzer assay. cfDNA was extracted from plasma leftovers collected after EV isolation by CB, UC or SEC. Internal standards are at 35bp and 10380bp. Plasma aliquots (1.8mL) were from HD #PMD1.58.

EV: Extracellular Vesicles; **CB**: Charge – Based EV Isolation Method; **UC**: Ultracentrifugation; **SEC:** Size Exclusion Chromatography; **dsDNA**: double-stranded DNA; **EV-RNA**: RNA samples extracted from EV; **cfDNA**: cell free DNA; **HDs**: Healthy Donors.

Supplementary Figure 2 related to Main Figure 1. ONCE protocol, combined with CB, allows efficient isolation of EV and cfDNA from plasma of blood collected into EDTA or Streck tubes.

- **a.** Scheme of ONCE protocol performed by applying the CB method for EV isolation. ONCE protocol is the sequential isolation of EV and cfDNA from the same aliquot of diluted plasma. I CONTROL (I-CTRL) is the original method for cfDNA isolation from a single aliquot of not–diluted plasma. II CONTROL (II – CTRL) is the isolation of the cfDNA from a single aliquot of diluted plasma. Plasma dilution is an essential step to reduce plasma viscosity thereby facilitating the binding of the beads to the EV.
- **b.** Quantification of EV by TRPS measurements. EV were isolated by CB from plasma of blood collected in K2EDTA tubes. EV were from a cohort of n=20 HDs.
- **c.** Quantification of cfDNA obtained by Qubit dsDNA HS assay (Thermo Fisher Scientific). cfDNA samples were extracted from plasma leftover collected after EV isolation. Data are from n=20 HDs. An aliquot of blood was collected from each individual donor in K2EDTA tubes and processed for plasma separation. The volume of plasma was split into three aliquots. Each aliquot was processed with a dedicated protocol (either one of ONCE, I-CTRL or II- CTRL II) as shown in **panel a**. n.s.: not significant differences by One – way Anova.
- **d.** Quantification of EV by TRPS measurements. EV were isolated by CB from plasma of blood collected in Streck tubes. EV were from a cohort of n=20 HDs.
- **e.** Quantification of cfDNA by Qubit dsDNA HS assay (Thermo Fisher Scientific). cfDNA was extracted from plasma leftovers collected after EV isolation. Samples are from a cohort of n=20 HDs. An aliquot of blood was collected from each individual donor in Streck tubes and processed for plasma separation. The volume of plasma was split into three aliquots and each of them was processed with a dedicated protocol (either one of ONCE, I-CTRL or II- CTRL II) as shown in **panel a**. n.s.: not significant differences by One – way Anova.
- **f.** Representative profiles of cfDNA obtained by Agilent HS DNA bioanalyzer assay. cfDNA samples were extracted from plasma of blood collected into K2EDTA tubes. Samples were processed according to protocols ONCE, I- CTRL, II- CTRL as shown in panel A. All profiles are comparable demonstrating that the length of fragments of cfDNA isolated from diluted plasma after EV recovery (**ONCE**) is comparable to cfDNA obtained from not diluted plasma (**I- CTRL**) or from diluted plasma without processing for EV isolation (**II-CTRL**). The typical cfDNA fragment size (about 174-176 bp) is evident across all profiles. Internal standards are at 35bp and 10380bp.
- **g.** Representative profiles of cfDNA obtained by Agilent HSDNA bioanalyzer assay. cfDNA samples were extracted from plasma of blood collected into Streck tubes. Samples were processed according to protocols ONCE, I- CTRL, II- CTRL as shown in panel A. All profiles are comparable demonstrating that the length of fragments of cfDNA isolated from diluted plasma after EV recovery (**ONCE**) is comparable to cfDNA obtained from not diluted plasma (**I- CTRL**) or from diluted plasma without processing for EV isolation (**II-CTRL**). The typical cfDNA fragment size (about 174-176 bp) is evident across all profiles. Internal standards are at 35bp and 10380bp.

ONCE: ONe Aliquot for Circulating Elements; **EV:** Extracellular Vesicles; **cfDNA**: cell free DNA; **HDs**: Healthy Donors; **TRPS**: Tunable Resistive Pulse Sensing; **CB**: Charge – Based EV Isolation Method; **I- CTRL**: First Control Protocol; **II-CTRL**: Second Control Protocol; cfDNA: cell free DNA, **K2EDTA tube**: tube containing di-potassium K2EDTA which blocks the coagulation cascade. K2EDTA tubes are

commonly used for examination of whole blood in haematology. **STRECK tube**: Cell-Free DNA BCT STRECK is a blood collection tube that which stabilizes nucleated blood cells preventing release of genomic DNA and allowing isolation of high-quality cell-free DNA. Cell-Free DNA BCT tubes (cfDNA BCTs) are commercialized by Streck (La Vista, NE).

Supplementary Figure 3 related to Main Figure 2. Small EV isolated from BrCa patients at early stage contain minuscule amount of dsDNA.

- **a.** Western Blot assay showing the EV-enriched proteins Tsg101, Flotillin 2, Flotillin 1, CD9 and the contaminant proteins Apolipoprotein A1 (ApoA1) and Albumin in representative n=3 HER2+ and n=3 HER2- EV from BrCa patients (PMB). EV were isolated by CB isolation method as shown on **Fig. 1a**.
- **b.** Quantification of EV-associated dsDNA by Qubit dsDNA HS assay. dsDNA was extracted from EV after isolation by CB. EV were from early stage or metastatic BrCa patients. Synthetic liposomes (100-200nm diameter size) containing artificial dsDNA (DNA-encapsulating liposomes) were utilized as internal control. The concentration of dsDNA recovered from early stage BrCa patients is miniscule, not suitable for ddPCR and/or sequencing assays.
- **c.** Quantification of EV samples utilized for dsDNA extraction. Measurements were performed by NTA. EV were isolated from plasma aliquots of BrCa patients at early stage (n=3) or advanced, metastatic stage (n=2). The concentration of EV is significantly higher on samples derived from metastatic patients. * p value= 0.0112 by unpaired t-test.
- **d.** Representative EV-DNA profiles obtained by Agilent HS DNA bioanalyzer assay. Samples are EV from plasma of metastatic or early stage BrCa patients or synthetic liposomes containing artificial dsDNA (internal control). Yellow arrows indicate signals associated to DNA.
- **e.** Profiling of sequenced EV-RNA samples by Agilent RNA 6000 Pico bioanalyzer assay. Internal standard is at 25bp. Labels report BrCa patient ID and subtype. All EV-RNA were extracted from EV isolated by CB from plasma of early stage BrCa (PMB).
- **f.** Profiling of sequenced cfDNA samples by Agilent HS DNA bioanalyzer assay. Labels report BrCa patient ID and subtype. All cfDNA were isolated in the framework of ONCE from plasma leftover of early stage BrCa (PMB). Internal standards are at 35bp and 10380bp.

EV: Extracellular Vesicles; **EV-DNA**: DNA extracted from EV; **EV-RNA**: RNA extracted from EV; **dsDNA:** double-stranded DNA; **BrCa:** Breast Cancer; **NTA:** Nanoparticle Tracking Analysis.

Supplementary Figure 4 related to Main Figure 3. HER2 protein is carried by EV.

- **a.** Western Blot assay showing the breast tissue associated proteins HER2, CD44, EpCaM and EV-enriched proteins Flotillin 1, Flotillin 2 and CD9 in representative n=2 protein extracts of EV samples isolated by CB from conditioned medium of SKBR3 (HER2+) and MDAMB231 (HER2-) human breast cancer cell lines.
- **b.** Quantification of PE+ signal detected by imaging flow cytometry (Amnis Imagestream^x MK II). EV samples were stained with the IgG isotype control (PE-Conjugated) for the anti-HER2 antibody utilized in **Fig. 3c**. As expected, only minimal and comparable percentages of fluorescent particles are detected on both HER2- and HER2+ samples. Data are from technical replicates of measurements performed on EV samples isolated from n=3 HER2- and n=3 HER2+ BrCa patients.

Supplementary Figure 5 related to Main Figure 4. Tissue based ERBB2 status of the TCGA BrCa cohort

a. Plot derived from tissue based sequencing data of BrCa patients deposited on the cancer genome atlas (TCGA) data portal. X axis: *ERBB2* Number of copies by DNA-Seq; Y axis: expression of *ERBB2* gene as quantified by RNA-Seq datasets/studies (Ciriello G, Gatza ML, Beck AH, Wilkerson MD, Rhie SK, Pastore A, Zhang H, et al. Comprehensive Molecular Portraits of Invasive Lobular Breast Cancer. Cell 2015;163:506-19). Specifically, data from RNA-Sequencing and DNA Sequencing were analyzed to evaluate how DNA- associated or RNA – associated information predict the HER2 Status (positive vs negative). Red and blue colors show stratification of BrCa by IHC tissue biopsy (protein – associated data). Marginal boxplots show distributions for each group of samples. Horizontal and vertical dotted lines indicate thresholds to define *ERBB2* gain or over-expression.
