# Supplementary material for: Integrating extracellular vesicle and circulating cell‐free DNA analysis using a single plasma aliquot improves the detection of HER2 positivity in breast cancer patients
Source: J Extracell Biol. 2023 Sep 25;2(9):e108. doi: 10.1002/jex2.108 (PMC10688391; doi:10.1002/jex2.108)
Supplement: Supplementary file 3 — Supporting Information [file JEX2-2-e108-s003.docx]

**Supplementary Materials and Methods**

**Experimental design and human sample collection**

Healthy donors (n=20; males and females, age >18 years old with no medical history of cancer or anti-cancer treatments) prospectively enrolled on a voluntary basis on a protocol approved by the University of Trento Ethics Committee (ID # 2017-010). Healthy donor plasma samples were used to test diverse EV isolation methods, followed by cfDNA extraction within the ONCE framework.

Breast cancer patients’ plasma samples were obtained from a cohort of patients prospectively enrolled on a protocol approved by the Ethics Committee of Santa Chiara Hospital in Trento (Rep.Int.12315 of July 24, 2017) with written informed consent. Eligibility criteria included breast cancer diagnosis and recommendations for neoadjuvant therapies. A set of n=44 patients to similarly represent each of the four most common subtypes of breast cancer ((HER2+, Luminal B (HER2+), Luminal B (HER2-), TNBC)) was initially selected to compare the circulating tumor–derived content (EV and cfDNA) from HER2 positive and HER2 negative breast cancer samples at diagnosis. To then ensure a large enough set of patients with adequate EV-RNA and cfDNA yields for the ddPCR assays, additional patients were selected from the same prospectively collected clinical cohort (ddPCR successfully performed on n=38 BrCa patient plasmas), and n=7 commercially available healthy donor plasma samples (males and females, age > 40 years old with no medical history of cancer or anti-cancer treatments) were purchased from Precision Medicine Group, LLC as controls.

**ONCE Protocol: combined isolation of EV and cfDNA from human plasma**

*EV isolation by Charge-Based (CB) method and cfDNA extraction*: The human plasma samples were filtered (Minisart NML syringe filters, pore size: 0.8 µm; Sartorius) and diluted with 1x Phosphate-Buffered Saline without calcium and magnesium buffer (Gibco) at 1:3 (v/v) ratio and processed for EV isolation by a charge – based isolation method as described before (Notarangelo et al., 2020; Notarangelo et al., 2019). The diluted plasma was recovered and processed for cfDNA isolation by QIAmp Circulating Nucleic Acid kit (Qiagen) (Lee et al., 2018).

To verify the efficiency of cfDNA extraction after EV isolation by CB method, we collected blood from n=20 HDs. Two types of tubes commonly utilized for liquid biopsies, i.e., K2EDTA and BCT Streck tubes, were used. Each volume of the separated plasma sample was split into three aliquots (1.5ml/each). The three aliquots of plasma were then processed, each with one of the three following protocols as described in **Supplementary** **Fig. 2a**:

*i)* ONCE Protocol, the plasma aliquot was diluted in PBS1X and incubated with EV-capture beads for EV isolation; the diluted EV-depleted plasma leftovers are then utilized for cfDNA extraction.

*ii)* I CONTROL protocol, the aliquot was processed to purify cfDNA according to the protocol commonly utilized for cfDNA isolation (Chen et al., 2020);

*iii)* II CONTROL protocol, the aliquot is first diluted in PBS1X (as in *i*) and then processed to purify cfDNA, as in *ii*, to check whether the sole dilution of the plasma (performed to reduce plasma viscosity) interferes with the efficiency of cfDNA recovery.

*EV isolation by ultracentrifugation (UC) and cell-free DNA (cfDNA) extraction:* EV were separated by UC on an Optima MAX-XP ultracentrifuge (Beckman Coulter) equipped with a TLA55 rotor. The plasma samples were filtered (Minisart NML syringe filters, pore size: 0.8 µm; Sartorius) and cleaned from cell debris by two serial centrifugation steps: 2000g for 10min and 10.000g for 20min. EV were then pelleted at 100.000g for 70min, washed with 1mL of 1xPBS (Gibco), and re-pelleted at 100.000g for 70min as previously reported (Thery et al., 2006). The liquid fractions (plasma and washing PBS) recovered from the UC steps were pooled in a separate clean tube and processed for cfDNA isolation as before (Lee et al., 2018).

*EV isolation by Size Exclusion Chromatography (SEC) (Izon qEV2 columns) and cfDNA extraction:* Plasma samples were filtered (Minisart NML syringe filters, pore size: 0.8 µm; Sartorius) and cleaned from debris by two serial centrifugation steps: 1.500g for 10 min followed by 10.000g for 10 min before loading on a pre-rinsed qEV2/70 nm column (Izon Science LTD, Cat. No. SP4). EV were collected from 1^st^ to 5^th^ fractions by using an Izon Automatic Fraction Collector (AFC) and concentrated in Amicon Ultra 15 filtering units (Merck Millipore, Cat. No. UFC910024) by centrifugation at 3.000g for 20 min on a 5810R benchtop centrifuge (Eppendorf). The volumes eluted between the 6^th^ and the 20^th^ fractions were collected and processed for cfDNA isolation as before (Lee et al., 2018).

**EV isolation by Charge-Based (CB) method from cell culture media (CCM)**

The SKBR3 and MDA-MB-231 cell lines were cultured in McCoy’s 5A and DMEM medium (26600-023, 11960-044, Gibco), respectively. The media were supplemented with 10% FBS (10270-106, Gibco), 1% Glutamine (25030-024, Gibco), and 1% Pen/Strep (30-002-CI, Corning). Before collecting CCM, the cells were washed two times with 10 mL of PBS 1X (Gibco) and maintained for 24h in regular medium without FBS. The CCM was processed to remove cell debris with two sequential centrifugations (300g x 10 minutes, 2800 rpm x 10 minutes) and filtered (Minisart NML syringe filters, pore size: 0.2 µm; Sartorius). Afterward, the CCM was concentrated with Amicon Ultra centrifugal devices MWCO 100 kDa (Merck) for 10 minutes at 3000g. EV were isolated from concentrated CCM by the CB method as previously described (Notarangelo et al., 2020).

**Quantitation of EV**

The size and concentration of the EV isolated from BrCa patients were quantitated using Tunable Resistive Pulse Sensing (TRPS, qNANO instrument, Izon Science). An average of 2 min recording time was used with nanopores NP250 (A58579, A68549; Izon Science). Voltage was set between 0.30 and 0.40 to achieve and maintain a stable current in the 95-130 nA range, noise between 7-12pA and a linear particle count rate. Calibration was performed using a known concentration of beads CPC200B (mode diameter: 210nm) or CPC400E (mode diameter: 340nm; Izon Science). All the acquisition data were recorded and analyzed by Izon Control Suite v.3 software. Based on instrumentation availability, size distribution profiles and concentration of the EV isolated from healthy donors’ plasma were obtained using Nanoparticle Tracking Analysis (NTA, NanoSight NS300, Malvern Panalytical). All EV samples were diluted 1:40-1:2000 in 1x Phosphate-Buffered Saline without calcium and magnesium buffer (Gibco) to obtain between 20 and 120 particles /frame. For each sample, 5x 60 s videos were recorded in standard mode with the equipped sCMOS camera (camera level 15-16) and analyzed by NTA 3.4 software (Malvern Panalytical) with a detection threshold ranging from 3 to 5.

**DNA Extraction from EV**

DNA was extracted from EV-enriched samples by using DNeasy Blood and Tissue kit (Qiagen; cat.69504) as this methodological approach was previously reported for studies on DNA cargo of EV (Vagner et al., 2018). We followed the manufacturer’s instructions with a few modifications: EV were resuspended in 200uL of PBS1x before adding 20uL of provided Proteinase K, 4uL of RNAseA (Qiagen; cat. 19101, 100ug/mL) and 200uL of AL buffer (without ethanol). Samples were incubated at 56°C for 10min, mixed with 200uL of 100% ethanol (Sigma-Aldrich) to obtain a homogeneous solution, and pipetted into a DNeasy mini spin column. Afterward, columns were washed with 500uL of AW1 and AW2 buffers and dried at room temperature for 2 min. EV-DNA was eluted by adding 40uL of 10mM Tris-HCl ph8.0 and centrifuging columns at 6.000g for 2min. Measurements of DNA quantity were performed on 10uL of eluted EV-DNA by High Sensitivity dsDNA Qubit Assay (Thermo Fisher Scientific).

**DNA-encapsulating liposomes**

DNA-encapsulating liposomes utilized as internal positive control were prepared by the hydration of a thin lipid film. Briefly, 220 μL of 40 mg/mL POPC (1-palmitoyl-2-oleoyl-glycerol-3-phosphocholine; Avanti Polar Lipids) and ten mol% cholesterol (0433-250G, VWR Chemicals) in chloroform (288306, Sigma-Aldrich) were deposited in a 3 mL glass round bottom flask, and the solvent evaporated with a Buchi rotary evaporator. The thin lipid film was kept under vacuum for two hours before DNA encapsulation. Plasmid DNA (1.3 μg, 4Kb) was dissolved in sterile PBS1x (Gibco) and then added to the lipid film and thoroughly vortexed for four min. 5 freeze-thaw cycles were performed to increase encapsulation efficiency. The liposomes were then transferred to 1 mL glass vials and tumbled overnight at RT on a tube rotator unit (10136-084, VWR). After tumbling, the liposomes were extruded through 400 nm Whatman Nuclepore track-etched polycarbonate membranes with an Avanti Mini-Extruder (11 passes) and purified by size exclusion chromatography with Sepharose 4B resin (GE17-0120-01, Cytiva) equilibrated with PBS1x (Gibco). Fractions were collected with a Gilson FC204B multichannel fraction collector. The purified liposomes were utilized within a few hours.

**RNA Extraction from EV-enriched samples**

EV-RNA was isolated by adding TRIzol Reagent (Invitrogen) and chloroform to samples. The RNA-containing phase was separated by centrifugation (10.000 rpm x 15min), mixed with isopropanol (Sigma-Aldrich), and incubated with glycogen (Thermo Fisher Scientific, RNA grade) at -20ºC overnight. RNA was precipitated by centrifugation (12.000 rpm x 40min at 4ºC) and washed with 70% ethanol by two sequential centrifugation steps (12.000 rpm x 20min; 12.000 rpm x 20min). Dried RNA pellets were further purified with a Single Cell RNA Purification Kit (Norgen; cat.51800). EV-RNA were analyzed on Agilent 2100 Bioanalyzer G2939A coupled with 2100 Expert version 2.6 software.

**Quantitation of cfDNA and bioanalyzer analysis**

The size distribution of cfDNA fragment length was analyzed by Agilent High Sensitivity DNA Kit on Agilent 2100 Bioanalyzer G2939A and quantitated by Qubit® dsDNA HS (High Sensitivity) Assay Kit (Thermo Fisher Scientific, Inc.).

**Quantitation of EV-RNA and bioanalyzer analysis**

RNA extracted from EV was analyzed for size distribution and quantitation of fragments length by Agilent RNA 6000 Pico Assay or Agilent Small RNA Assay on Agilent 2100 Bioanalyzer G2939A.

**Digital PCR**

Copy Number Determination Assay was performed on cfDNA according to ddPCR Supermix for probes (No dUTP) (Bio-Rad Laboratories Inc.; #1863024). Specifically, 2x ddPCR ddPCR Supermix for probes (No dUTP), 20x target probe (FAM) and 20x reference probe were loaded to 1x as final concentration in a final volume of 22uL. Template (cfDNA) was loaded in a final volume of 6uL without prior digestion with restriction enzymes. Cycling conditions were: 95°C for 10 min, 39 cycles of 94°C for 30 sec and 60°C for 1min followed by 98°C for 10 min and holding at 4°C until droplets reading.

Gene expression Assay was performed on EV -RNA according to the One-Step RT-ddPCR Advanced Kit for Probes (Bio-Rad Laboratories, Inc.; #1864021). Reaction mix was prepared according with the manufactures’ protocol in a final volume of 22uL. Supermix, 20x target probe (FAM) and 20x reference probe were loaded to 1x as final concentration. Reverse transcriptase was 20U/uL, except in negative control wells. Reverse transcription was performed at 42°C for 60 min and immediately followed by 95°C for 10 min to activate enzyme. Denaturation and Annealing/Extension were 40 cycles of 95°C for 30 sec and 59-60°C for 1 min. enzyme was deactivated at 98°C for 10 min and PCR reactions hold at 4°C until reading. Probes for *ERBB2* (dHsaCP1000116; dHsaCPE5037554), EIF2C1 (dHsaCP2500349), EEF2 (dHsaCPE5050049) were from Bio-Rad Laboratories Inc. Amplification on cfDNA was determined as the ratio between circulating ERBB2 and reference gene EIF2C1 by using a previously reported ddPCR assay for ERBB2 copy number alteration on BrCa tissues (Tantiwetrueangdet et al., 2018). The expression on EV-RNA was calculated as a ratio between the detection of fragments corresponding to ERBB2 and the commonly utilized tissue reference gene EEF2 (Ersahin et al., 2014). PCR reactions were run on T100 thermal cycler (Bio-Rad, Hercules, CA, USA). ddPCR reaction conditions were verified using varied amounts of DNA, RNA and EV-RNA extracted from HER2+ and HER2- cell lines. Reactions without template and /or enzymes were run as internal negative controls. Droplets were generated by QX200 AutoDG Droplet Digital PCR System, and PCR plates were analyzed on a Bio-Rad QX200 droplet reader (Bio-Rad Laboratories, Inc.). Analysis of ddPCR data was analyzed using QuantaSoft Software, version 1.7 (Bio-Rad Laboratories, Inc.).

**EV protein isolation and Western blot analysis**

EV proteins were extracted and analyzed by western blotting as previously reported (Choi et al., 2020) with few modifications: total EV proteins were loaded on 4-15% mini – PROTEAN TGX stain-free gels (Bio-Rad), transferred on a trans-blot turbo system (Bio-Rad) and visualized on a UVITEC imaging system (UVItec Ltd, Cambridge). Primary antibodies used were rabbit anti -CD9 (#D801A; Cell signaling Technology; 1/500); rabbit anti – CD 9 ( # MA5-33125; Invitrogen; 1/500); mouse anti – Apolipoprotein A1 (#5F4; Cell Signaling Technology; 1/250); rabbit anti – Albumin (#4929; Cell Signaling Technology; 1/1000), mouse anti-Tsg-101 (ab83, Abcam, 1/1000), rabbit anti-Flotillin 1 (#18634, Cell Signaling Technology; 1/1000), rabbit anti- Flotillin 2 (#3436, Cell Signaling Technology; 1/1000), rabbit anti-CD44 (ab157107, Abcam, 1/500), rabbit anti-EpCam (#D1B3, Cell Signaling Technology; 1/500), rabbit anti-HER2/ErbB2 (#29D8, Cell Signaling Technology; 1/1000).

**Imaging Flow Cytometry**

Isolated EV samples (10^10^-10^11^ particles/mL) were resuspended in 1x Phosphate-Buffered Saline (1xPBS) (Gibco) and incubated overnight at 4C with HER2/ErbB2 (29D8) – PE-conjugated antibody (1:50) or concentration-matched Rabbit (DA1E) mAb IgG XP® Isotype Control (PE Conjugate) (Cell Signaling Technology). EV were then stained with one volume of CellMask plasma membrane stains (C10046, Life Technologies) diluted in 1xPBS (1:5000) for 15 minutes at room temperature, in the dark. Before acquisition on Amnis ImageStream X MkII, the stained samples were diluted with 1xPBS to 10^8^ EV / mL to avoid coincidence.

All samples were analyzed on an Amnis ImageStream X MkII (Luminex). All data sets were acquired with a 60x objective on High Gain mode using INSPIRE® instrument acquisition software (Luminex). Fluidics was set to low speed; sensitivity was set to high resolution. Samples were acquired with all lasers run at maximal power (488nm:200mW, 642nm:150mW, 758nm:70mW). Sub-micron beads (F13839, Life Technologies) were run after instrument initialization as an internal standard. Double-stained EV samples from BrCa patients were loaded on tubes and acquired for the same time (3 minutes). To avoid the carryover of fluorophores and dyes during the acquisition, washes with deionized water (0.1 μm filtered) were performed between samples. The following control samples were run before double stained samples as internal controls: i) 1x Phosphate-Buffered Saline (PBS) without calcium and magnesium buffer (Gibco) only; ii) unstained EV; iii) staining solutions (1x PBS plus CellMask or PE-conjugated antibodies).

**Multiplex EV surface marker analysis**

Isolated EV (5*10^8^ - 10^9^ particles counted by NTA) were analyzed for surface protein expression using the MACSPlex Exosome kit (Miltenyi Biotech; no. 130-108-813) by following the manufacturers’ protocol tube for overnight capture. Detection was performed by incubating samples with 15μL of the provided MACSPlex Exosome Detection Reagent cocktail for 2 hours at room temperature. All samples were run as triplicates, and PBS (without EV) was included as blank control. Data were acquired on a BD FACSCanto flow cytometer (BD Biosciences) and analyzed with FlowJo v10 Software (BD Life Sciences). Normalized and background-corrected MFI values were used for sample comparison.

***In situ* analyses of breast cancer patient clinical cohort**

Fluorescence in situ hybridization (FISH) assay for ERBB2 was performed using the ZytoLight CEN17/SPEC ERBB2 Dual Color Probe (ZytoVision) by following the ZytoLIGHT Implementation KIT for in situ hybridization. Slides were analyzed on a Zeiss Axio-Imager 2 according to ASCO-CAP guidelines (Wolff et al., 2018). Immunohistochemistry staining was performed as part of the clinical laboratory workflow per guidelines. All *in situ* analyses were performed at the Unit of Surgical Pathology of the Santa Chiara Hospital in Trento, Italy.

**DNA whole-exome sequencing**

For library preparation (SeqCap EZ HyperCap Workflow version 2.3; Roche), we utilized 20-50ng of cfDNA and 100ng of matched germline DNA (gDNA) sonicated to reduce the size to 180-220bp (Covaris M220). Libraries were sequenced on Illumina HiSeq2500 platform by the Next Generation Sequencing Facility at the University of Trento (Italy) with a Paired-End, 100bp protocol with a mean coverage of 537x (min=282x, max=731x) for cfDNA and of 96x for gDNA (min=89x, max=109x) (**Supplementary Table 4**). Fastq files were controlled for quality by fastqc (www.bioinformatics.babraham.ac.uk) and trimmed by Trimmomatic (Bolger et al., 2014) (SLIDINGWINDOW:4:15 MINLEN:36). Alignment was performed using BWA (Li and Durbin, 2009) with default parameters. Duplicate removal, realignment around indels, and base quality recalibration were performed with MarkDuplicates, RealignerTargetCreator, IndelRealigner, BaseRecalibrator and ApplyBQSR from gatk4 pipeline (Van der Auwera et al., 2013). The genetic match of cfDNA and control gDNA was verified by SPIA (Demichelis et al., 2008). CNVkit (Talevich et al., 2016) was used for copy number aberration (CNA) detection via segmentation, and Log2 values of cfDNA over control were corrected for purity and ploidy using ClonetV2 (Prandi and Demichelis, 2019). Results were visualized using IGV (Thorvaldsdottir et al., 2013) and custom R scripts (libraries: circlize (Gu et al., 2014)). See also **Supplementary Table 2**.

**RNA-Seq**

To generate libraries for RNA-Seq, the purified EV-RNA were processed with SMART-Seq® Stranded Kit (Takara Bio USA, Inc.). Libraries were sequenced on the Illumina HiSeq2500 platform by the Next Generation Sequencing Facility at the University of Trento (Italy) with a Single-End, 100bp Single-End protocol generating an average of 88M reads (min=56, max=97) (**Supplementary Table 5**). Fastq files were controlled for quality and trimmed using fastp (Chen et al., 2018). Reads were aligned against the human genome (hg38) using STAR (Dobin et al., 2013) (custom parameters, outFilterMatchNminOverLread 0.3, outFilterScoreMinOverLread 0.3).

Breast cancer tissue data (Ciriello et al., 2015) were downloaded from CBioPortal (<https://www.cbioportal.org/>). Capped relative linear copy-number values were used for DNA. RNA Seq V2 RSEM was used for RNA. Clinical data annotation includes immunohistochemistry evaluation of HER2.

**HER2 positivity classification of plasma and tissue samples**

For HER2 positivity classification of BrCa study patients, we used ddPCR data for all liquid biopsy samples (the highest DNA and RNA ddPCR values of healthy donor samples were used as lower thresholds for DNA and RNA, respectively) and sequencing data for the TCGA tissue samples (DNA amplification threshold was set at 2.6 copies; RNA levels for samples without DNA amplification (DNA copies < 2.6) were considered and the 75% percentile of the distribution was set as lower threshold).

For each sample (either plasma or tissue), we then considered the following classes of HER2+ positivity; *Only RNA*: RNA but not DNA signal is higher than the corresponding threshold; *Only DNA*: DNA but not RNA data is higher than the corresponding threshold; *Combo AND*: both DNA and RNA data are above the corresponding thresholds; *Combo OR*: any of DNA or RNA data is higher than the corresponding threshold.

For classificatory accuracy estimation, precision is defined as:

$$precision= \frac{true positives}{true positive+false positives}$$

Recall (i.e., sensitivity) is defined as:

$$recall= \frac{true positives}{true positives+false negatives}$$

Specificity is defined as:

$$specificity= \frac{true negatives}{true negatives+false positives}$$

F1 score is defined as:

$$F1= \frac{2*precision*recall}{precision+recall}$$

All performance measures of plasma and tissue-based HER2 positivity are shown in **Supplementary Table 5**.

References

Bolger, A.M., Lohse, M., and Usadel, B. (2014). Trimmomatic: a flexible trimmer for Illumina sequence data. Bioinformatics *30*, 2114-2120.

Chen, S., Zhou, Y., Chen, Y., and Gu, J. (2018). fastp: an ultra-fast all-in-one FASTQ preprocessor. Bioinformatics *34*, i884-i890.

Chen, Z., Sun, T., Yang, Z., Zheng, Y., Yu, R., Wu, X., Yan, J., Shao, Y.W., Shao, X., Cao, W.*, et al.* (2020). Monitoring treatment efficacy and resistance in breast cancer patients via circulating tumor DNA genomic profiling. Mol Genet Genomic Med *8*, e1079.

Choi, D., Go, G., Kim, D.K., Lee, J., Park, S.M., Di Vizio, D., and Gho, Y.S. (2020). Quantitative proteomic analysis of trypsin-treated extracellular vesicles to identify the real-vesicular proteins. J Extracell Vesicles *9*, 1757209.

Ciriello, G., Gatza, M.L., Beck, A.H., Wilkerson, M.D., Rhie, S.K., Pastore, A., Zhang, H., McLellan, M., Yau, C., Kandoth, C.*, et al.* (2015). Comprehensive Molecular Portraits of Invasive Lobular Breast Cancer. Cell *163*, 506-519.

Demichelis, F., Greulich, H., Macoska, J.A., Beroukhim, R., Sellers, W.R., Garraway, L., and Rubin, M.A. (2008). SNP panel identification assay (SPIA): a genetic-based assay for the identification of cell lines. Nucleic Acids Res *36*, 2446-2456.

Dobin, A., Davis, C.A., Schlesinger, F., Drenkow, J., Zaleski, C., Jha, S., Batut, P., Chaisson, M., and Gingeras, T.R. (2013). STAR: ultrafast universal RNA-seq aligner. Bioinformatics *29*, 15-21.

Ersahin, T., Carkacioglu, L., Can, T., Konu, O., Atalay, V., and Cetin-Atalay, R. (2014). Identification of novel reference genes based on MeSH categories. PLoS One *9*, e93341.

Gu, Z., Gu, L., Eils, R., Schlesner, M., and Brors, B. (2014). circlize Implements and enhances circular visualization in R. Bioinformatics *30*, 2811-2812.

Lee, H., Na, W., Park, C., Park, K.H., and Shin, S. (2018). Centrifugation-free extraction of circulating nucleic acids using immiscible liquid under vacuum pressure. Sci Rep *8*, 5467.

Li, H., and Durbin, R. (2009). Fast and accurate short read alignment with Burrows-Wheeler transform. Bioinformatics *25*, 1754-1760.

Notarangelo, M., Ferrara, D., Potrich, C., Lunelli, L., Vanzetti, L., Provenzani, A., Basso, M., Quattrone, A., and D'Agostino, V.G. (2020). Rapid Nickel-based Isolation of Extracellular Vesicles from Different Biological Fluids. Bio-protocol *10*, e3512.

Notarangelo, M., Zucal, C., Modelska, A., Pesce, I., Scarduelli, G., Potrich, C., Lunelli, L., Pederzolli, C., Pavan, P., la Marca, G.*, et al.* (2019). Ultrasensitive detection of cancer biomarkers by nickel-based isolation of polydisperse extracellular vesicles from blood. EBioMedicine *43*, 114-126.

Prandi, D., and Demichelis, F. (2019). Ploidy- and Purity-Adjusted Allele-Specific DNA Analysis Using CLONETv2. Curr Protoc Bioinformatics *67*, e81.

Talevich, E., Shain, A.H., Botton, T., and Bastian, B.C. (2016). CNVkit: Genome-Wide Copy Number Detection and Visualization from Targeted DNA Sequencing. PLoS Comput Biol *12*, e1004873.

Tantiwetrueangdet, A., Panvichian, R., Wongwaisayawan, S., Sueangoen, N., and Lertsithichai, P. (2018). Droplet digital PCR using HER2/EIF2C1 ratio for detection of HER2 amplification in breast cancer tissues. Med Oncol *35*, 149.

Thery, C., Amigorena, S., Raposo, G., and Clayton, A. (2006). Isolation and characterization of exosomes from cell culture supernatants and biological fluids. Curr Protoc Cell Biol *Chapter 3*, Unit 3 22.

Thorvaldsdottir, H., Robinson, J.T., and Mesirov, J.P. (2013). Integrative Genomics Viewer (IGV): high-performance genomics data visualization and exploration. Brief Bioinform *14*, 178-192.

Vagner, T., Spinelli, C., Minciacchi, V.R., Balaj, L., Zandian, M., Conley, A., Zijlstra, A., Freeman, M.R., Demichelis, F., De, S.*, et al.* (2018). Large extracellular vesicles carry most of the tumour DNA circulating in prostate cancer patient plasma. J Extracell Vesicles *7*, 1505403.

Van der Auwera, G.A., Carneiro, M.O., Hartl, C., Poplin, R., Del Angel, G., Levy-Moonshine, A., Jordan, T., Shakir, K., Roazen, D., Thibault, J.*, et al.* (2013). From FastQ data to high confidence variant calls: the Genome Analysis Toolkit best practices pipeline. Curr Protoc Bioinformatics *43*, 11 10 11-11 10 33.

Wolff, A.C., Hammond, M.E.H., Allison, K.H., Harvey, B.E., Mangu, P.B., Bartlett, J.M.S., Bilous, M., Ellis, I.O., Fitzgibbons, P., Hanna, W.*, et al.* (2018). Human Epidermal Growth Factor Receptor 2 Testing in Breast Cancer: American Society of Clinical Oncology/College of American Pathologists Clinical Practice Guideline Focused Update. J Clin Oncol *36*, 2105-2122.
